# Exercise addiction: A review and evaluation of current research and theory

**DOI:** 10.1556/2006.2025.00336

**Published:** 2026-04-22

**Authors:** Bhavya Chhabra, Zsolt Demetrovics, Mark D. Griffiths, Attila Szabo

**Affiliations:** 1 Institute of Health Promotion and Sport Sciences, Faculty of Education and Psychology, ELTE Eötvös Loránd University, Budapest, Hungary; 2 Doctoral School of Education, Faculty of Education and Psychology, ELTE Eötvös Loránd University, Budapest, Hungary; 3 Flinders University Institute for Mental Health and Wellbeing, College of Human Sciences and Culture, Flinders University, Bedford Park, SA, Australia; 4 Institute of Psychology, ELTE Eötvös Loránd University, Budapest, Hungary; 5 Centre of Excellence in Responsible Gaming, University of Gibraltar, Gibraltar; 6 International Gaming Research Unit, Psychology Department, Nottingham Trent University, 50 Shakespeare Street, Nottingham, UK; 7 Faculty of Health and Sport Sciences, Széchenyi István University, Győr, Hungary

**Keywords:** behavioral addiction, addictive behavior, exercise addiction, compulsive exercise, dysfunction, epidemiology, etiology, prevention, treatment, assessment, compulsive behavior, impulsive behavior, comorbidity

## Abstract

**Background and Aims:**

While regular physical activity provides many health benefits, exercise can cause more harm than good when done excessively to the point where a person loses control. This condition has been called various names, but the most accurate term is ‘exercise addiction’ (EA) because it reflects both compulsive behavior and dependence. EA is classified as a non-substance-related addictive disorder, or behavioral addiction, and has been the subject of research for over fifty years. However, it is not officially recognized in major diagnostic systems, mainly due to significant conceptual and measurement challenges. This paper provides an overview of current knowledge on EA, including its causes, assessment issues, epidemiology, associated conditions, negative effects, and options for treatment and prevention.

**Methods:**

An evaluation-driven narrative review was conducted which synthesized findings from empirical research and theoretical models adopted across EA research. It critically examined prevailing conceptualizations, methodological challenges, and potential treatment approaches.

**Results:**

Epidemiological findings are inconsistent and report inflated prevalence rates, partly due to reliance on self-report instruments that often fail to distinguish pathological exercise from passion. Etiological models emphasize the complex interplay between biological, psychological, and behavioral factors. Negative consequences include physical injury, emotional distress, and social strain. Evidence for effective interventions remains sparse.

**Discussion and Conclusions:**

Despite growing interest, progress remains slow. There is a need for larger, longitudinal, and experimental studies to advance understanding. Though not included in the DSM-5, greater clinical awareness is vital for early detection and prevention. EA is clinically relevant but still poorly defined, requiring robust empirical and theoretical work.

## Introduction

Physical inactivity has been identified as the fourth major risk factor and a significant contributor to premature deaths worldwide (Olave et al., 2025; World Health Organization [WHO], 2023). It is also linked to various health issues, including dementia, type 2 diabetes, cardiovascular diseases, strokes (WHO, 2024), respiratory problems, and an increased risk of all-cause mortality (Katzmarzyk, Friedenreich, Shiroma, & Lee, 2022). Additionally, it contributes to the development of specific cancers such as breast, ovarian, bowel, uterine, colorectal, and endometrial cancers (Australian Institute of Health & Welfare, 2017) and colon cancer (WHO, 2024). In contrast, regular and *optimal* exercise, defined as planned, structured, and organized set of repeated movement activities, carried out with optimal intensity, regulated frequency, and duration, has many benefits and adds to overall health promotion (Caspersen, Powell, & Christenson, 1985; Huang, Huang, & Wu, 2022). These benefits include enhanced brain and mental health (Gori, Topino, & Griffiths, 2024; Huang et al., 2022; WHO; 2024), reduced risk of nearly 40 chronic diseases (Ruegsegger & Booth, 2018; Wang et al., 2024), and overall good quality of life (Huang et al., 2022; Warburton & Bredin, 2019; WHO, 2024).

Paradoxically, exercise can shift from a protective behavior to a maladaptive one. In some cases, when personal and situational factors interact, exercise can become dysfunctional (Dinardi, Egorov, & Szabo, 2021; Egorov & Szabo, 2013). Exercising obsessively or beyond an individual's control (Bulgay et al., 2025), whether to manage stress or enhance performance, can result in compulsive behavior and consequent harm (Egorov & Szabo, 2013). What begins as health- or performance-driven activity may turn problematic, leading individuals to progressively increase intensity or duration to achieve the same benefits. This escalating cycle can result in a loss of control over training volume, posing serious risks to both physical and mental health (Szabo, 2010; Szabo & Demetrovics, 2022). This maladaptive form of exercise is often termed ‘exercise addiction’ (EA) (Bulgay et al., 2025; Wang et al., 2025).

The conceptual complexity (Szabo, Griffiths, de La Vega Marcos, Mervo, & Demetrovics, 2015), multidimensional nature (Sicilia, Paterna, Alcaraz-Ibanez, & Griffiths, 2021), inconclusive research results (Sicilia, Alcaraz-Ibáñez, Paterna, & Griffiths, 2023), and substantial growth of research on the topic over the past 25 years, justifies the adoption of a narrative review approach to critically appraise and synthesize the current body of knowledge on EA. This method is timely because EA requires a broad exploration, comprehensive overview, and evaluative critical synthesis. Moreover, the evaluation-driven framework adopted in the present review supports the need for nuanced interpretation along with fair critique (Furley & Goldschmied, 2021; Sukhera, 2022). Therefore, the present paper employed a focused and an evaluative narrative review approach to critically appraise the central methodological inconsistencies in the field, examining the construct's classification as a psychological disorder, thereby informing its status on inclusion in medical reference nosologies. It further investigates and discusses the key factors required for the proper arbitration of EA as a clinical dysfunction. Accordingly, the present review explores EAs phenomenological features, related definitions and terminology, its epidemiological characteristics, etiology, strengths and limitations of different assessment approaches, co-occurring disorders, physical and mental health consequences, the available preventive and treatment approaches, and offers recommendations for future research directions.

## Definition and symptomology

### Origins

Over half a century ago, the concept of EA emerged when Baekeland (1970) studied sleep in regularly exercising students. The findings showed that during periods of exercise deprivation, individuals experienced disrupted sleep, increased anxiety, and sexual tension. At that time, exercise was not termed as an ‘addiction’ but as a way to alleviate aggressive tendencies, consistent with the catharsis theory of stress (Szabo, Tóth, Kósa, Laki, & Ihász, 2021). Exercise improves self-esteem. However, when interrupted, it can cause increased urges, such as sexual desires, which, when fulfilled, help to relieve internal pressure.

Later, the concept of ‘positive addiction’ emerged (Glasser, 1976), highlighting the beneficial effects of exercise and its positive impact on health. These positive addictions primarily included exercise and meditation because they were viewed as self-improvement activities, and empowered individuals with resilience and support. Moreover, they were seen as activities that could be used to overcome more traditional substance use-related disorders. Glasser was inspired by Roger Kahn's book on professional baseball *The Boys of Summer* (1972), specifically George Schuba's discipline in baseball batting. Schuba recalled swinging his baseball bat 200–300 times, going to bed, and then getting back up because he could not sleep until he completed 600 full swings. This led Glasser to view Schuba's behavior as an addiction because of his compulsion to swing his baseball bat and because he experienced withdrawal symptoms if he was unable to do so.

Glasser recognized that this practice helped Schuba in major baseball leagues, and consequently, he labeled it as a positive addiction (Glasser, 1976). Morgan (1979) raised concerns about Glasser's idea, noting that excessive exercise could also result in physical harm or injury. He identified this behavior as a potential genuine form of addiction and considered it to be a behavioral dysfunction (i.e., a negative addiction, differing from Glasser's concept of positive addiction). In the same year, Sachs and Pargman (1979) defined EA as involving both psychological and physical dependence, using the term ‘running addiction’ to describe the associated withdrawal symptoms. Later, building on Morgan's critique, Griffiths (1996) asserted that Glasser's six criteria for positive addiction (e.g., must be non-competitive and needing about an hour a day, involve no self-criticism, easy to be done alone, no mental effort required, believed to have some value, believed that if persisted in will lead to improvement) did not align with recognized components of addiction (e.g., withdrawal, salience, conflict, mood modification, relapse, tolerance).

### Clarifying definitions

Exercising pathologically, characterized by a loss of control with excessive amount of time spent training, leads to physical, psychological, and/or social harm, which can result in EA (Szabo & Demetrovics, 2022). It is often accompanied by cravings or a constant urge for more exercise (Szabo & Demetrovics, 2022). The same condition has also been conceptualized using various names, and often used interchangeably, owing to serious ambiguity (Berczik et al., 2012; Szabo et al., 2015; Trott et al., 2021). The terms include: ‘exercise dependence’ (Schaub et al., 2024; Xie et al., 2024), ‘compulsive exercise’ (Con et al., 2024; Harris et al., 2024), ‘excessive exercise’ (Cosh, Eshkevari, McNeil, & Tully, 2023), ‘pathological exercise’ (Coniglio, Davis, Sun, Loureiro, & Selby, 2023; Cunningham, Pearman III, & Brewerton, 2016), ‘obligatory exercise’ (Guo et al., 2023; Wu et al., 2023), ‘problematic exercise’ (Barker, Kolar, Lazzer, & Keel, 2022; Sicilia, Alcaraz-Ibáñez, Paterna, & Griffiths, 2022), ‘maladaptive exercise’ (Schaumberg, Bulik, & Micali, 2023, 2024), ‘exercise abuse’ (Calogero & Pedrotty, 2004; Davis, 2000), ‘morbid exercise’ (Alcaraz-Ibáñez et al., 2020, 2021), ‘dysfunctional exercise’ (Fernandez-del-Valle, Quesnel, Mitchell, & Robert-McComb, 2023; Quesnel, Cooper, Cook, & Calogero, 2025), and ‘obsessive exercise’ (Boone, 1990). Moreover, different terms are also often employed within the same study, such as, ‘exercise addiction’ and ‘obligatory exercise’ (Popat, Dinu, Runswick, Findon, & Dommett, 2021) or ‘exercise addiction’ and ‘compulsive exercise’ (Cook, Hausenblas, & Freimuth, 2014).

**Fig. 1.**
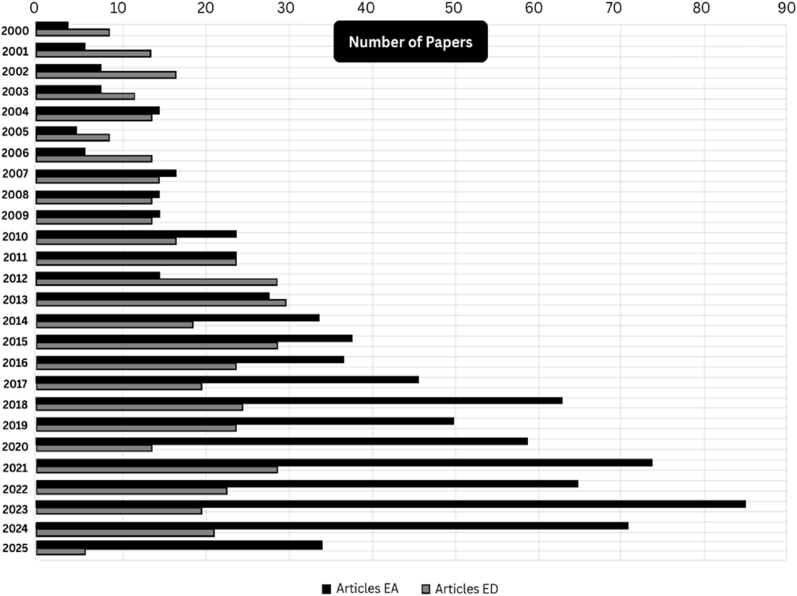
Research papers with “exercise addiction” (EA) and “exercise dependence” (ED) in their titles, published between 2000 and July 2025, identified for each year on *Google Scholar* utilizing the “advanced search” function (updated from Szabo, 2024)

Based on the *advanced search* function of *PubMed* and *Google Scholar* (see Table 1), the terms ‘exercise addiction’ and ‘exercise dependence’ are the two most used terms in academic literature, with the term ‘exercise addiction’ being more commonly used than ‘exercise dependence’ (see Fig. 1). While dependence does not fully capture the complexity of addiction, EA is most applicable because addiction encompasses both dependence and compulsion in describing uncontrolled exercise (Berczik et al., 2012; Mónok et al., 2012; Szabo & Demetrovics, 2022)*.* The general formula is *“addiction = dependence + compulsion”* (Szabo & Demetrovics, 2022, p. 70). This is further supported by the components model of addiction (Griffiths, 2005), which posits that all addictions comprise six core components (i.e., salience, mood modification, tolerance, withdrawal, conflict, and relapse). Meyer et al. (2025) and Ahorsu et al. (2023) further advocate that the term ‘exercise addiction’ is warranted because it exhibits clinical features that closely parallel substance addictions, such as persistence despite negative consequences and withdrawal-like symptoms. These similarities reinforce its clinical relevance and its value for guiding future interventions.

**Table 1.** Number of papers employing different terminologies for exercise addiction

Terms used in the title of published papers (across two scholastic databases)	Number of studies identified on *PubMed* (until July 2025)	Number of studies identified on *Google Scholar* (until July 2025)
Exercise addiction	130	758
Exercise dependence	117	547
Compulsive exercise	96	254
Excessive exercise	68	209
Pathological exercise	18	32
Obligatory exercise	13	61
Problematic exercise	9	20
Maladaptive exercise	11	21
Exercise abuse	2	10
Morbid exercise	3	4
Dysfunctional exercise	2	11
Obsessive exercise	0	5
More than one of the above	4	21

### Lack of diagnostic criteria

**Fig. 2.**
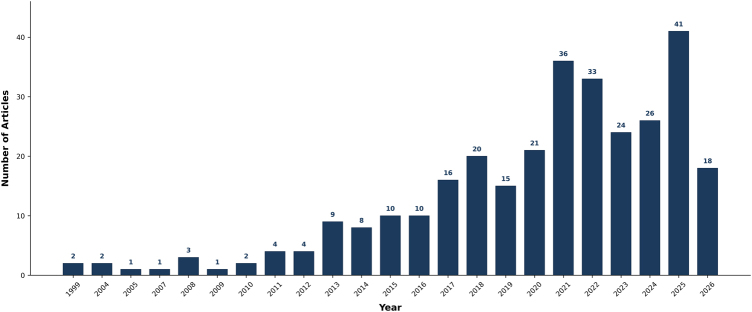
The rise in scientific research on exercise addiction (EA) over the last decades, as indicated by a search of titles and abstracts in the PubMed medical database

Research on EA has markedly increased, primarily since 2019 (see Fig. 2), with growing concern over its potential health risks. However, EA is not formally recognized within any official clinical diagnostic framework (Alcaraz-Ibáñez et al., 2020; Çakın et al., 2021). For example, neither the fifth (text-revised) edition of the *Diagnostic and Statistical Manual of Mental Disorders* (DSM-5-TR; American Psychiatric Association, 2022) nor the 11th revision of the *International Classification of Diseases* (ICD-11; World Health Organization [WHO], 2019/2021) include EA as a mental health disorder (Chhabra, Nazlıgül, & Szabo, 2024; Szabo, 2024; Weinstein & Szabo, 2023). This exclusion largely reflects persistent conceptual problems and insufficient scientific evidence for EA's etiological and symptomatologic patterns, which are essential to establish the related course descriptions and diagnostic criteria (Sicilia et al., 2022; Szabo & Demetrovics, 2022; Szabo & Kovacsik, 2019). Moreover, there are very few (i) nationally representative epidemiological surveys, (ii) neurobiological studies, (iii) studies with clinical samples, and (iv) treatment/intervention studies. Szabo (2024) further indicated that most evidence points to EA being symptomatic of another disorder, which may be a further reason for its exclusion in DSM-5. Close evaluation of comorbidities in EA is therefore crucial.

### Exercise addiction as a behavioral addiction

EA is increasingly recognized as a behavioral addiction (Gjoneska, Bőthe, Potenza, Szabo, & Demetrovics, 2024; Gori et al., 2024; Szabo & Demetrovics, 2022). Such addictions are characterized by compulsive engagement in non-substance-related behaviors that are perceived as relieving, though difficult to control (Brand et al., 2022; Gjoneska et al., 2024; Potenza, 2017). This behavioral pattern often results in clinically significant psychological distress or functional impairment (Brand et al., 2022). It progressively assumes priority in the individual's daily life and continues despite serious negative consequences across multiple life domains, warranting clinical attention (Brand et al., 2025; see ‘Negative consequences’ subsection below).

Cognitive and affective impairments frequently associated with behavioral addictions typically include poor decision-making, cue-reactivity, deficits in executive functioning, and reduced inhibitory control (Brand et al., 2025). Unlike substance-use disorders, behavioral addictions involve ordinary behaviors, performed with indulgence to the extent of self-harming (Szabo & Demetrovics, 2022). In essence, three core mechanisms underlie and maintain behavioral addictions (Brand et al., 2025):

- Persistent urges driven by positive or negative reinforcement of the behavior.
- Habitual engagement fueled by compulsive motivation and preoccupation with the behavior.
- Diminished self-control despite awareness of harmful consequences.

### Phenomenology of exercise addiction

In the context of EA, excessive engagement in exercise, marked by loss of behavioral control, increased priority (salience), mood regulation, withdrawal effects, tolerance, intrapersonal or interpersonal conflicts, and relapse mirror the clinical criteria used in behavioral addictions research (Chhabra, Árok, & Szabo, 2024; Griffiths, 2005; Szabo & Demetrovics, 2022). Exercise addiction involves compulsive exercise behavior that exceeds healthy limits (Minutillo et al., 2024). It is often accompanied by dependence resulting in adverse health, professional, and social consequences (Szabo et al., 2015). It can also lead to physical injuries and financial consequences (Hausenblas & Downs, 2002).

When the mood-enhancing effects of exercise are short-lived, addicted exercisers may exhibit key characteristics of EA. These include emotional distress, sleep disturbances, feelings of deprivation during inactivity, guilt due to a missed exercise session, low appetite, constant urges, prolonged exercise duration, persistence despite physical injury, apathy in other activities, and personal discomfort (Abrantes et al., 2019; Kalayasiri & Rattanawijarn, 2025; Minutillo et al., 2024). A key distinguishing characteristic of EA—compared to healthy physical activity—is the inability to refrain from exercising despite serious negative consequences which impair day-to-day functioning (Gori et al., 2024; Szabo, Griffiths, & Demetrovics, 2019). What begins as healthy can become harmful. The following sections in the present narrative review elaborate more on this complex phenomenon.

### Primary and secondary exercise addiction

When excessive exercise becomes maladaptive, it is often framed in terms of whether it is a primary issue or secondary to another disorder. If exercise itself is the main problem (e.g., as an escape response from stress), it is classified as *primary exercise addiction* (Berczik et al., 2012). However, if it (co-)occurs alongside other disorders, such as anorexia nervosa, bulimia nervosa, or various body-image dysfunctions, it is referred to as *secondary exercise addiction* (Bulgay et al., 2025; Godoy-Izquierdo, Ramírez, Díaz, & López-Mora, 2023). The main distinction between the two lies in etiology and the focus. In primary exercise addiction, the reward is *directly* associated with exercise only (Weinstein & Szabo, 2023). In secondary exercise addiction, the primary goal is *weight loss*, with excessive exercise being the primary strategy for achieving that (Berczik et al., 2012; Szabo & Demetrovics, 2022). Additionally, there are other conditions where EA is secondary to another psychological condition and has nothing to do with weight loss, such as obsessive-compulsive disorders, body dysmorphic disorder (Meyer et al., 2021), or muscle dysmorphia (Foster, Shorter, & Griffiths, 2015). Therefore, in secondary EA, the reward is *indirectly* tied to exercise fulfillment, and excessive exercise is *instrumental* in attaining another separate goal (Weinstein & Szabo, 2023).

## Assessment

It is important to note that there is currently no formal clinical diagnosis for EA (Weinstein & Szabo, 2023). Instead, the field is progressing through the development of various psychometric assessment instruments that evaluate problematic forms of exercise. Over the past 40 years, more than 30 instruments have been created to assess this problematic behavior in English. Table 2 briefly describes the different psychometric properties of these assessment tools and their underlying conceptual frameworks. Many of these tools assess dysfunctional attitudes and behaviors related to exercise training, ranging from low (asymptomatic) to high (symptomatic) levels of problematic exercise (Weinstein & Szabo, 2023). Some tools, such as the Negative Addiction Scale (NAS; Hailey & Bailey, 1982), the Excessive Exercise Scale (EES; McCabe & Vincent, 2002), and the Exercise Salience Scale (ESS; Kline, Franken, & Rowland, 1994), are rarely used in EA research (Berczik et al., 2012; Szabo & Demetrovics, 2022). Conversely, the Exercise Addiction Inventory (EAI; Terry, Szabo, & Griffiths, 2004) and the Exercise Dependence Scale (EDS; Hausenblas & Downs, 2002) are among the most widely used tools. However, the former is preferred over the latter because of its addiction symptoms specificity, brevity, and simplicity in scoring (Mónok et al., 2012; Szabo & Demetrovics, 2022).

**Table 2.** *Characteristics of psychometric instruments assessing exercise addiction (EA*)

Instrument	Author (year; newest first)	Type	No. of Items	No. of Subscales	Factors Identified	Theoretical Underpinnings	Empirical Underpinnings	Comments
1. Exercise Addiction Inventory (third version) (EAI-3)	Granziol et al. (2023)	6-point Likert Scale	8	2	Salience; mood modification; withdrawal; conflict; tolerance; relapse; Addictive tendencies (AT); Health relevance (HR)	Components model of addiction (Griffiths, 2005)	EFA, CFA; Cronbach's alpha: *α* = 0.81	
2. Secondary Exercise Addiction Scale (SEAS)	Trott, Johnstone, McDermott, Mistry, and Smith (2022)	6-point Likert Scale	11	2	Exercise addiction; eating disorders	Based on Brown's (1997) general components of addiction and DSM-5 criteria for eating disorders	CFA; Cronbach's alpha: *α* = 0.85	Used to assess EA and eating disorders
3. Revised Exercise Addiction Inventory (EAI-R)	Szabo, Pinto, Griffiths, Kovacsik, and Demetrovics (2019)	6-point Likert Scale	6	1	Salience; mood modification; withdrawal; conflict; tolerance; relapse	Components model of addiction (Griffiths, 2005)	CFA; Cronbach's alpha: *α* = 0.90	
4. Exercise Addiction Scale (EAS)	Demir, Hazar, and Cicioğlu (2018)	5-point Likert Scale	17	3	Excessive focus and emotion change; postponement of individual-social needs and conflict; tolerance development and passion			
5. Exercise Addiction Inventory – Youth Version (EAI-Y)	Lichtenstein, Griffiths, Hemmingsen, and Støving (2018)	5-point Likert Scale	6	1	Salience; conflicts (with parents over excessive exercise); mood modification; tolerance; withdrawal symptoms; conflict (academics and job); relapse	Six components of behavioral addictions (Griffiths, 2005)	Cronbach's alpha: *α* = 0.70	
6. Compulsive Exercise Test-4 Factors (CET-4F)	Swenne (2016)	6-point Likert Scale	21	4	Avoidance & rule driven behavior; weight control exercise; mood improvement; lack of exercise enjoyment			
7. Problematic Practice of Physical Exercise (PPPE)	Kotbagi, Kern, Romo, and Pathare (2015)	6-point Likert Scale	25		Lack of control; stereotypical behavior; motivation for (physical and psychological) health; withdrawal, interference with social life; tolerance	No distinct conceptual framework provided		
8. Compulsive Exercise Test- Athletes (CET-A)	Plateau et al. (2014)	6-point Likert Scale	15	3	Avoidance of negative affect; weight control exercise; mood improvement			
9. Exercise Dependence and Elite Athletes Scale (EDEAS)	McNamara and McCabe (2013)	5-point Likert Scale	24	6	Unhealthy eating behavior; conflict and dissatisfaction; more training; withdrawal; emotional difficulties; continuance behavior	DSM-IV substance dependency criteria	Factor analysis; Cronbach's alpha: *α* = 0.82	Specific for elite athletes
10. Obligatory Exercise Questionnaire – Revised (OEQ-R)	Duncan et al. (2012)	4-point Likert Scale	10	3	Preoccupation with exercise; exercise behavior; exercise emotionality	Problematic exercise as a behavior to manage body weight and shape	EFA, CFA	
11. Compulsive Exercise Test (CET)	Taranis, Touyz, and Meyer (2011)	6-point Likert Scale	24	5	Avoidance and rule-driven behavior; weight control exercise; mood improvement; lack of exercise enjoyment; exercise rigidity	Problematic exercise used to control body weight and shape; Cognitive, behavioral and emotional aspects	Cronbach's alpha: *α* = 0.72–0.88	Used in EA and eating disorders' research
12. Commitment to Physical Activity Scale-Revised (CPA-R)	DeBate, Huberty, and Pettee (2009)	4-pointScale	12	3	Value of physical activity; attitudes toward physical activity; motivation for physical activity	Problematic exercise behaviors exist at one end of the exercise spectrum	Chronbach's alpha: *α = *0.82–0.84	
13. Exercise Addiction Inventory (EAI)	Terry et al. (2004)	5-point Likert Scale	6	1	Salience; mood modification withdrawal; conflict; tolerance; relapse	Based on Brown's (1997) general components of addiction and components model of addiction (Griffiths, 2005)	Cronbach's alpha: *α* = 0.84	Widely adopted; Most concise psychometrically validated instrument in EA research
14. Exercise Dependence Scale-Revised (EDS-R)	Downs, Hausenblas, and Nigg (2004)	6-point Likert Scale	21	7	Tolerance; withdrawal; intention effects; lack of control; time; reduction in other activities; continuance	DSM-IV criteria for substance dependence	Cronbach's alpha: *α* = 0.78–0.95	
15. Exercise Commitment Survey (ECS)	Garman, Hayduk, Crider, and Hodel (2004)	Self-report	23		Frequency; duration; intensity of physical activity; commitment to exercise			
16. Excessive Exercise Scale (EES)	McCabe and Vincent (2002)	5-point scale	8	2	Focus on exercise; need for exercise	Problematic exercise as a behavior to manage body weight and shape	EFA; Cronbach's alpha: *α* = 0.86	Designed for adoloscents
17. Exercise Dependence Scale (EDS)	Hausenblas and Downs (2002)	6-point Likert Scale	30	7	Tolerance; withdrawal; intention effects; lack of control; time; reduction in other activities; continuance	DSM-IV criteria for substance dependence	CFA; Cronbach's alpha: *α* = 0.78–0.92	Widely used
18. Obligatory Exercise Questionnaire (OEQ-2)	Ackard, Brehm, and Steffen (2002)	4-point Likert Scale	11	3	Exercise fixation; exercise frequency; exercise commitment	Problematic exercise as a behavior to regulate body weight and shape	Factor analysis	Assesses some aspects of problematic exercise and body image disturbance
19. Obligatory Exercise Questionnaire (OEQ-1)	Steffen and Brehm (1999)	4-point Likert Scale	10	3	Emotional element of exercise; exercise frequency and intensity; exercise preoccupation	Problematic exercise as a behavior to regulate body weight and shape		
20. Bodybuilding Dependence Scale (BDS)	Smith, Hale, and Collins (1998)	7-point Likert Scale	9	3	Social dependence training dependence; mastery dependence	DSM-IV criteria for substance dependence	For the three subscales: Cronbach's alpha: *α* = 0.78, 0.76, 0.75 respectively	Exclusive to bodybuilding
21. Exercise Beliefs Questionnaire (EBQ)	Loumidis and Wells (1998)	Rating scale (0–100)	21		Social desirability; physical appearance; mental and emotional functioning; vulnerability to disease and aging	Problematic exercise as a dependence	For the four subscales: Chronbach's alpha: *α* = 0.87, 0.83, 0.89, 0.67 respectively	
22. Exercise Dependence Questionnaire (EDQ)	Ogden, Veale, and Summers (1997)	7-point Likert Scale	29	8	Interference with social/family/work life; positive reward; withdrawal symptoms; exercise for weight control, insight into problem; exercise for social reasons, exercise for health reason; stereotyped behavior	Exercise dependence may be primary or secondary to eating disorders, or a unitary construct (Veale, 1987; Yates, Leehey, & Shisslak, 1983), and is linked to mood regulation processes (Morgan, Costill, Flynn, Raglin, & O’connor, 1988)	Cronbach's alpha: *α* = 0.84	
23. Exercise Salience Scale (ESS) (a)	Kline et al. (1994)	5-pointScale	40	2	Response omission anxiety; Response persistence + four minor unclear factors	No distinct conceptual framework	Factor analysis	Rarely employed
24. Commitment to Exercise Scale (CES)	Davis, Brewer, and Ratusny (1993)	Visual analogue scale	8	One dimensional scale	Pathological aspects of exercise; obligatory aspects of exercise	Problematic exercise as end of a continuum of exercise; Item generation was based on evaluation of published case studies on pathological exercise (e.g., Yates et al., 1983).	Factor analysis; Cronbach's alpha: *α* = 0.77	
25. Compulsive Exercise Scale	Tuttle (1992)						Cronbach's alpha: *α* = 0.88	
26. Running Addiction Scale (RAS)	Chapman and De Castro (1990)	Self-rated addiction scale; 5-point Likert Scale	11			Run in spite of obstacles and withdrawal effects	Cronbach's alpha: *α* = 0.82	Specific to runners
27. Running Addiction Scale	Rudy and Estok (1989)	3-point Likert Scale	17				Cronbach's alpha: *α* = 0.66	
28. Obligatory Exercise Questionnaire (OEQ)	Pasman and Thompson (1988)	4-pointScale	20		1 factor	Problematic exercise as a behavior to manage body weight and shape		
29. Commitment to Physical Activity Questionnaire (CPA)	Corbin, Nielsen, Borsdorf, and Laurie (1987)	5-pointScale	12		Unidimensional structure	Problematic exercise behaviors exist at one end of the exercise spectrum		
30. Obligatory Running Questionnaire (ORQ)	Blumenthal, O’Toole, and Chang (1984)	Rating: true or false	21					
31. Negative Addiction Scale (NAS) (Portuguese adapted version)	Modolo et al. (2011)	0 = absence of a symptom 1 = presence of a symptom; cutoff score of 5 points	14			Based on Negative Addiction Scale (NAS); (Hailey & Bailey, 1982)	Cronbach's alpha: *α* = 0.79	Focus on ‘negative’ psychological effects of dependence

*Note.* DSM = Diagnostic and Statistical Manual; EFA = exploratory factor analysis; CFA = confirmatory factor analysis.

English records (on *Google Scholar)* were searched using the following key terms: “exercise addiction”, “exercise dependence”, “compulsive exercise”, “excessive exercise”, “pathological exercise”, “obligatory exercise”, problematic exercise”, maladaptive exercise”, “exercise abuse”, “morbid exercise”, “dysfunctional exercise”, and “obsessive exercise.

### Strengths and limitations of psychometric instruments

The psychometric assessment tools with a strong theoretical framework demonstrate high internal consistency, which is crucial for EA research. However, recent meta-analytic evidence from 255 studies (Alcaraz-Ibáñez, Paterna, Sicilia, & Griffiths, 2022a) reported pooled Cronbach's alpha values ranging from 0.76 to 0.93 across major self-report measures of problematic exercise. These findings, influenced by sociodemographic and methodological factors, underscore inconsistencies in measurement rigor and the need for more systematic reliability evaluation. The interpretation of Cronbach's alpha cut-offs has also been questioned for relying on researchers' subjective judgment rather than empirical evidence (Hoekstra, Vugteveen, Warrens, & Kruyen, 2019). Moreover, Alcaraz-Ibanez, Paterna, Griffiths, and Sicilia (2024b) reported that key instruments assessing problematic exercise lacked methodological rigor and adequate validity and reliability. Although the translation and validation across cultural contexts have improved the accessibility of these instruments (Khoshro et al., 2024; Szabo, Griffiths, & Demetrovics, 2019), some adapted versions exhibit reduced reliability, indicating limitations in linguistic equivalence and the need for more robust cross-cultural validation (Alcaraz-Ibáñez et al., 2022a). Overall, the inconsistent definitions and operational components further complicate the understanding of this complex phenomenon (Sicilia et al., 2021, 2022).

Scores on many of the problematic exercise screening tools usually suggest a level of risk of exercise addiction (REA). However, high scores may not necessarily materialize into dysfunction as they may share a covariance with passion, perfectionism, or sports commitment (Chhabra et al., 2025; Szabo, 2024; Szabo & Demetrovics, 2022). Chhabra et al. (2025) asserted the need to redirect focus from poorly defined *risk* construct to the rigorous study of *exercise addiction* itself. The boundary between healthy involvement in behavior and pathological behavior can often blur, requiring a more accurate evaluation (Hadzlik et al., 2024). That said, a score of 24 on the EAI (Terry et al., 2004) or 29 on the Revised Exercise Addiction Inventory (EAI-R; Szabo, Griffiths, & Demetrovics, 2019) could be seen as a potential warning sign. However, these scales are not intended for diagnostic purposes and are only suitable for surface-level screening (Chhabra, Granziol, et al., 2024; Szabo & Demetrovics, 2022).

### From assessment to practice

There remains a disconnect between clinical practice and academic research. Cases identified with high REA through these screening tools, should be followed up by clinical interviews (Chhabra, Granziol, et al., 2024; Szabo & Demetrovics, 2022). Szabo (2001) proposed the *pyramid approach* for this essential collaboration between researchers and clinical experts in the assessment and management of EA. At the base of this pyramid, trained researchers can conduct the surface screening of EA using psychometric assessment tools. Individuals identified at-risk of EA can then be referred to clinicians for detailed clinical interviews (middle layer of the pyramid) to understand what underlies the high score on the instrument. Finally, the top layer differentiates those who control their exercise from those who show maladaptive exercise patterns, with a focus on diagnosing co-morbidities and offering essential treatment (Szabo, 2024).

## Epidemiology

In assessing EA, various contradictory results have been reported. The primary reasons for these inconsistencies are multifaceted including the psychometric assessment tools employed (Sicilia et al., 2022), the type of physical activity (Lichtenstein, Melin, Szabo, & Holm, 2021; Mayolas-Pi et al., 2025), the characteristics of the studied sample (Godoy-Izquierdo, Ramírez, et al., 2023; Szabo et al., 2015), differences in how individuals subjectively interpret assessment items (across males and females from different cultures) (Griffiths et al., 2015; Szabo et al., 2015), and the varying sample sizes (Akbari et al., 2024; Berczik et al., 2012). For instance, one study (Beuno-Antequera et al., 2022), which included 330 Spanish cyclists, reported an EA prevalence of 6.1%, while another found a much higher rate of 15.4% among 317 Italian marathoners (Collado-Boira et al., 2021). Lichtenstein et al. (2021) reported 7.6% of EA prevalence among 417 elite Danish athletes, while Boeno-Antequera (2020) reported a higher EA prevalence of 13.3% among 1,014 Spanish indoor cyclists.

More recently, Chhabra, Granziol, et al. (2024) reported that 9.5% of 3,760 exercisers from 15 nations were at risk of EA. Higher risk was observed among athletes in organized sports and those motivated by mastery, a pattern also reported by Lichtenstein et al. (2021). In a study by Lassner et al. (2022), 6.94% of 72 German marathoners reported a risk of EA. Ahorsu et al. (2023) asserted that EA is typically prevalent in 3% to 13% among adolescents, and from 3% to 42% among those in the general exercising population. This substantial heterogeneity reflects notable methodological inconsistencies stemming from the variability in the screening tools used, the use of small self-selected convenience samples, and the type of exercise examined (Lichtenstein et al., 2021).

According to Di Lodovico, Poulnais, and Gorwood (2019), EA is most prevalent among endurance athletes (14%), followed by those engaged in team-based sports (10%), fitness-related (8%), and power sports (6%). The risk of EA also differs based on exercise involvement, whether in team or individual sports (Chhabra, Nazlıgül, & Szabo, 2024), and on cultural upbringing (Chhabra, Granziol, et al., 2024). For instance, Chhabra, Árok, and Szabo (2024) reported a more than fourfold higher prevalence of the risk of EA among Indian exercisers (21.86%; 485 participants) than among Hungarian exercisers (5.38%; 502 participants).

It is worth noting that EA could also be a plausible manifestation of eating disorders (EDs) given that EA is approximately 3.5 times more prevalent among individuals with EDs compared to those without (see ‘Co-occurring disorders’ section below; Akbari et al., 2024; Khoshro & Abbasalizad Farhangi, 2024; Trott et al., 2021).

### Gender differences

Regarding gender differences, a review of 27 studies on EA by Dumitru, Dumitru, and Maher (2018) concluded that males were more likely to be addicted to exercise than females. Similarly, in another review of 117 studies, Alcaraz-Ibanez, Paterna, Griffiths, Demetrovics, and Sicilia (2022b) reported that females more often reported problematic exercise symptoms for mood modification and weight control. In contrast, males reported such symptoms due to the harms caused by excessive over-involvement in exercise. Notably, assessment-related issues cause the variability in the gender related differences when understanding EA (Alcaraz-Ibanez et al., 2022b), highlighting a fundamental methodological concern.

Moreover, recent empirical research has also provided additional insight on this issue. Ganson, Lavender, Rodgers, Cunningham, and Nagata (2022) reported that 11% of males and 17% of females were at risk of developing EA. In contrast, Szabo et al. (2022), Strahler, Wachten, Stark, and Walter (2021), and Chhabra, Granziol, et al. (2024) found no gender differences in EA, with Strahler et al. reporting that the risk of EA was only slightly higher among males (4.9%) compared to females (4.7%).

### Do high prevalence figures genuinely indicate addiction?

The observed heterogeneity in the prevalence of EA may arise from various key factors (Godoy-Izquierdo, Ramírez, et al., 2023). First, clarity regarding the conceptual framework of EA remains limited, compounded by variability in the theoretical bases of assessment instruments (Berczik et al., 2012; Griffiths, Landolfi, & Szabo, 2023; Sicilia et al., 2022). Second, selecting a screening tool from a pool of instruments (Griffiths et al., 2015), further complicates the process of EA assessment instrument selection. Third, EA assessment instruments are limited to assessing the presence, susceptibility, intensity, or presumed *risk* of EA symptoms (Berczik et al., 2012) but they cannot be used to make a clinical diagnosis (Szabo, 2024). This limitation contributes to methodological confusion (Berczik et al., 2012; Szabo et al., 2015).

Fourth, because these instruments are not diagnostic tools (Szabo, 2018), findings based on them may inflate the prevalence estimates and risk of over-pathologizing EA (Weinstein & Szabo, 2023). The elevated scores likely signify factors beyond pathological tendencies or represent a strong passion or commitment to the sport (Szabo, 2018, 2024). Fifth, differences in the way scale items are subjectively interpreted by leisure exercisers versus elite athletes introduce additional conceptual challenges (Szabo et al., 2015). Lastly, the diversity in exercise types adds to the heterogeneity in EA prevalence (Colledge, Cody, et al., 2020; Griffiths et al., 2023). That said, screening for risk of EA in team sports may be futile because addictions typically do not arise in scheduled (team sport) behaviors. They are likelier only when athletes engage in *extra* individual training that is additional to their team sport exercise (Griffiths et al., 2023).

### Improving epidemiological evidence

Taken together, these issues significantly delay any credible pathway toward formal recognition of EA as a mental health disorder in texts such as the DSM and ICD. By focusing on prevalence estimates, the nomothetic approach offers little promise for advancing knowledge and over-pathologizes EA (Szabo, 2024). It is imperative to study *exercise addiction* itself, rather than relying on the uninformative metric of *risk* (Chhabra et al., 2025). True addiction to exercise is arguably rare (Juwono & Szabo, 2021). Even if only 0.5% of the exercising population experiences EA, it still represents a significant number of individuals in need of support (Berczik et al., 2012). Epidemiological data underscore the need for further rigorous high-quality research in this area (Berczik et al., 2012). Ideally, prevalence rates should rely only on clearly defined cases with EA (Weinstein & Szabo, 2023). According to the pyramid approach, collaboration between clinicians and academic researchers is critically important (Chhabra, Granziol, et al., 2024; Juwono & Szabo, 2021; Szabo, 2024), representing a potential paradigm shift in this research area.

## Co-occurring disorders

EA is commonly associated with psychiatric comorbidities such as EDs, anxiety, obsessive compulsive disorder, depression, substance use, attention deficit hyperactivity disorder (ADHD) (Colledge, Sattler, et al., 2020; Weinstein & Szabo, 2023), personality disorders (Colledge, Sattler, et al., 2020; Meyer et al., 2021), and social phobias (Gunnarsson et al., 2022). It often coexists with EDs, where compulsive exercise is used for weight control and mood regulation (Harris, Hay, & Touyz, 2020). In a meta-analysis, Trott et al. (2021) reported EA prevalence rates among individuals with EDs ranging from 29 to 80%. Research shows a clear relationship between EA, weight concerns, and body image issues (Godoy-Izquierdo, Ramírez, et al., 2023). Pioneering research by Yates et al. (1983) reported psychological similarities in body weight, exercise compulsion, and rigid eating patterns among both male and female runners with anorexia, with the same underlying pathology. Since then, several studies have recognized EA's overlap with disordered eating (Godoy-Izquierdo, Ramírez, et al., 2023). Some scholars argue that primary and secondary exercise dependence are distinct (Cook & Luke, 2017), while others believe EA is always secondary to EDs (Bamber, Cockerill, Rodgers, & Carroll, 2003; Godoy-Izquierdo, Ramírez, et al., 2023). Additionally, Kun et al. (2022) indicated that EDs may act as a contributing risk factor for EA, particularly in the presence of any psychological distress and emotion dysregulation. Conversely, deficits in emotion regulation could serve as a shared risk factor for both conditions, EA as well as ED, potentially explaining their high rate of comorbidity.

In addition to EA's well-documented association with EDs, EA has been implicated in a broader spectrum of psychopathology. Affected individuals frequently report elevated levels of anxiety and depression, often accompanied by pronounced feelings of guilt or irritability when unable to engage in exercise (Godoy-Izquierdo, Ramírez, et al., 2023; Harris et al., 2020). It has also been associated with substance -use disorders, specifically alcohol-use disorder (Ertl et al., 2022; Freimuth, Moniz, & Kim, 2011). Furthermore, growing evidence highlights the unsupervised consumption of image and performance-enhancing drugs (IPEDs), often informally referred to as lifestyle drugs, among individuals exhibiting addictive exercise behaviors (Dores et al., 2021). Research also indicates an association between EA and borderline personality traits (Maraz, Urbán, Griffiths, & Demetrovics, 2015). Psychological disturbances also often co-occur with EA, including increased neuroticism (Bircher, Griffiths, Kasos, Demetrovics, & Szabo, 2017; Kun et al., 2022) and a higher frequency of negative mood states (Sicilia, Alcaraz-Ibáñez, Dumitru, Paterna, & Griffiths, 2020). However, it remains unclear if EA is a distinct behavioral addiction or simply a common symptom of other psychiatric disorders (Weinstein & Szabo, 2023).

## Etiology

The theoretical models that attempt to explain EA broaden the understanding of this dysfunction. While they have conceptual similarities, they approach EA from different perspectives; nevertheless, they complement each other. Each model accounts for different aspects of EA, and despite their commonalities, they offer unique insights without being redundant. No single model is considered superior, rather they are simply distinct yet complementary (Szabo & Demetrovics, 2022). These models are generally categorized into three main groups: psychological, physiological, and behavioral, encompassing factors such as genetic, neurobiological, cognitive, psychological, social, personality, and cultural dimensions.

### Psychological models

#### Cognitive appraisal hypothesis

Szabo (1995) proposed the cognitive appraisal hypothesis which suggests that EA arises when a habitual exerciser begins to ‘depend’ on exercise for stress management after previously experiencing its stress-relief benefits. Therefore, if the individual cannot train due to an unexpected illness, injury, or an urgent chore, it is likely that they experience withdrawal, resulting in increased stress. Eventually, the addicted exerciser becomes trapped in a vicious cycle, needing more exercise each time they encounter stress, much of which can be caused by the exercising behavior itself.

#### Four-phase model

Freimuth et al. (2011) introduced the four phases of addiction, in which the exercise escalates from the first to the last phase—recreational, at-risk, problematic, and addictive—each defined by motivation, control, and consequences.

- Phase 1: Recreational exercise – This initial phase involves engaging in exercise recreationally and seeking pleasure, with an individual being motivated to achieve health-related goals. The behavior is under control and feels intrinsically rewarded with almost negligible adverse outcomes.
- Phase 2: At-risk exercise – In this phase, the focus shifts to exercising primarily for psychological benefits, particularly for mood modification. Motivation transitions from enjoyment of exercise to using it as a psychological escape, providing temporary relief from stress. This phase marks the onset of EA.
- Phase 3: Problematic exercise – Here, exercise becomes problematic when it starts to dominate daily life, with rigid exercise patterns taking precedence over other activities. Healthy involvement gradually transitions to a maladaptive form in this third phase. This causes negative physical and psychosocial consequences, withdrawal sets in, often leading to solitary exercise patterns with the individual struggling to maintain control over it.
- Phase 4: Exercise addiction – The final phase incorporates components such as salience, conflict, tolerance, mood modification, withdrawal, and relapse, and where an individual's whole life revolves around exercise (Griffiths, 2005).

#### Interactional model

Egorov and Szabo (2013) posited a person-specific model demonstrating how past experiences, life situations, personal values, and social images jointly influence whether individuals use exercise or other methods to cope with stress. These personal and situational interactions are numerous, and each case reflects a mental schema which is like a secret ‘black box’. Within this black box, there are subjective and complex mental processes that are unique to each individual. They represent the interactions between personal and situational life events. These interactions determine the motivation for exercise, which can be healthy and therapeutic (physical or psychological), and may also involve mastery and performance orientations. A crucial factor is the sudden reaction (e.g., relationship break-up, job loss, traumatic life event) that results from personal and situational interactions within this black box. This reaction arises in response to an overwhelming life stressor, causing psychological distress, and indicates that EA emerges abruptly rather than gradually, which is more *revolutionary* than *evolutionary* (Szabo, 2010). Due to the various interactions between psycho-situational factors, EA is best understood through idiographic analysis because each case is unique. This interactional model was recently expanded and revised by Dinardi et al. (2021), adding determinants such as self-concept and other stressors. In brief, it is posited that EA arises through two pathways: (i) therapeutic, linked to coping with hardships, and (ii) mastery, associated with ignoring physical limits which can lead to injury (Dinardi et al., 2021; Egorov & Szabo, 2013).

#### PACE (pragmatics, attraction, communication, expectation) model

Egorov and Szabo (2013) indicated that the aforementioned interactional model for EA aligns with the PACE model for addictions in general, which is why it has been described here following the interactional model. Sussman et al. (2011) posited the PACE (pragmatics, attraction, communication, expectation) model, a *general model* for all types of addictions. ‘Pragmatics’ refer to coping with overwhelming life stressors using available means, such as exercise. ‘Attraction’ refers to individual characteristics, situational factors, past experiences, and current exercise behaviors that influence the decision to exercise. ‘Communication’ refers to how interpersonal influences, beliefs, and thoughts shape exercise decisions. Finally, ‘Expectation’ refers to the higher expectations associated with exercise that reduce the likelihood of resorting to other forms of addiction.

#### Passion model

Lichtenstein, Jensen, Larsen, Omdahl, and Szabo (2020) posited the passion model of EA. In its hierarchical form, exercise is healthy and enjoyable to begin with, progressing to commitment and harmonious passion that reflects dedication and enjoyment. This can lead to discovery passion, which provides subjective rewards. This discovery of passion can be harmonious, but sometimes it can also become obsessive, for example, when an individual becomes obsessed with exercise. The critical factor is loss of control, which marks the transition from obsessive passion to EA, where psychobiological symptoms and negative consequences play a crucial role (Kovacsik et al., 2018; Szabo & Demetrovics, 2022).

### Physiological models

#### Biopsychosocial model

McNamara and McCabe (2012) proposed the biopsychosocial model, which highlights the development and maintenance of EA through the interplay of biological, psychological, and social factors. Biological factors, such as body mass index (BMI), interact with psychological factors, including the sense of self and exercise-related beliefs, and social factors, including the athlete's entourage, team dynamics, social support, and sociocultural pressures. These factors influence one another reciprocally, shaping the genesis and maintenance of EA, specifically among elite athletes.

#### Early physiological models

Exercise has a well-documented positive impact on mental health (Solmi et al., 2025; Čekić, 2024), demonstrating its substantial influence on the human psyche (Winiarz, 2019). Early research on EA theorized the ‘runner's high hypothesis,’ which is a sensation of euphoria (Dishman & O’Conner, 2009; Goldberg, 1988), characterized by reduced anxiety symptoms (Weiermair, Svehlikova, Boulgaropoulos, Magnes, & Eberl, 2024), inner harmony, joy (Nogueira, Molinero, Salguero, & Márquez, 2018), and decreased sensitivity to psychological pain (Weiermair et al., 2024). This euphoric state, caused by the release of endorphins, serves as a neurobiological reward and is linked to *β*-endorphin activity in the brain following aerobic activity (Nogueira et al., 2018). These endorphins are the body's natural opioid compounds (Winiarz, 2019). Previously, it was believed that *β*-endorphins drove neurogenesis (Winiarz, 2019), and that the activation of the opiate system caused the *runner's high* (Marshall & Goda, 2024). However, due to the large size of endorphins, they are prevented from crossing the blood-brain barrier and therefore cannot directly affect the brain.

This has led researchers to focus on endocannabinoids, natural compounds capable of crossing the blood-brain barrier to reduce anxiety and induce calmness, potentially explaining the post-run relaxed state (Linden, n.d.; Weiermair et al., 2024; Winiarz, 2019). Additionally, the brain and spinal cord have abundant cannabinoid receptors. During exercise, cannabinoids released by tissues bind to these receptors, alleviating pain and inducing a sense of euphoria (Marshall & Goda, 2024). According to Gillman, Hutchison, and Bryan (2015), long-distance runners often liken running to a “drug-like” experience and report withdrawal-like symptoms when unable to run. Therefore, the runner's high explains the genesis of EA.

The ‘sympathetic arousal hypothesis’ (Thompson & Blanton, 1987) suggests that regular exercise results in adaptations in the sympathetic nervous system, which leads to reduced sympathetic activity at rest. This reduction establishes a lower baseline level of arousal, which may feel insufficient for everyday activities, eventually causing a low sense of energy or lethargy. To counteract this, individuals may increase their exercise to boost arousal, resulting in addictive behavior. Additionally, the ‘catecholamine hypothesis’ (Cousineau et al., 1977) posits that exercise alters brain catecholamine activity, which plays a key role in regulating mood and the reward system. This may offer a potential explanation for the addictive nature of exercise.

### Behavioral models

Behaviorists suggest that addicted exercisers are driven to exercise because of negative reinforcement to avoid unpleasant outcomes and/or positive reinforcement (to experience the runner's high). The addicted exerciser also believes that if they do not indulge in the activity, something bad will happen to them (see ‘Negative consequences’ subsection below). Essentially, behavioral scientists argue that all human behavior can be understood through the lens of reinforcement and punishment (Berczik et al., 2012).

## Negative consequences

Addictive behavior is often described as obsessive, compulsive, and impulsive, with these tendencies showing little regard for possible adverse outcomes (Freimuth et al., 2011; Szabo & Demetrovics, 2022; Trott et al., 2021). The obsessive nature of this dysfunction involves increasing anxiety and restlessness before the behavior, with urges and cravings, followed by a psychological sense of relief once it is carried out (Berczik et al., 2012). This pattern is cyclic, as urges resurface repeatedly.

The resulting negative effects are psychological (such as feelings of powerlessness and inability to control behavior), physical, and social, or a combination of these (Alcaraz-Ibáñez, Paterna, Griffiths, & Demetrovics, 2024a; Berczik et al., 2012; Guo et al., 2025; Szabo, 2024). They can potentially lead to injury, reduced well-being, and in severe cases, even death or suicide (Godoy-Izquierdo, Navarrón, López-Mora, & González-Hernández, 2023). For example, an addicted runner might choose to keep running despite a serious injury, fully aware that it could cause further harm. As a result, these events can harm a person's social life, leading to interpersonal conflicts, apathy toward other activities, low mood, financial debts, poor academic performance due to an overwhelming focus on exercise (salience) (Szabo & Demetrovics, 2022; Weinstein & Szabo, 2023), and a decreased quality of life (Brand et al., 2022). In summary, EA leads to neglect of other important responsibilities, which can spiral into significant impairment across various areas of daily functioning (Ahorsu et al., 2023).

Additionally, such individuals often feel a compulsion to engage in physical activity, fearing the negative consequences. For them, exercise evolves from a mere personal desire (‘want’) into a burdensome obligation (‘I ought to’) that must be fulfilled (Szabo & Demetrovics, 2022). Beyond these negative consequences, the components model of addiction (Griffiths, 2005) is essential to identify a case with EA (Szabo & Demetrovics, 2022).

## Treatment

Partly due to the lack of established diagnostic criteria for EA, no specific treatment has been defined for the condition, leaving treatment efficacy unproven. While no controlled clinical trials currently exist for EA, a combination of intervention therapies with theoretical relevance may prove effective, depending on the severity of the addiction and the individual's motivation to change (Szabo & Demetrovics, 2022; Weinstein & Szabo, 2023). For individuals with moderate EA, psychoeducation that focuses on self-regulation (Asadi et al., 2021), emphasizing SMART goals (specific, measurable, achievable, realistic, and timely), along with information about the benefits and risks of exercise (Hausenblas, Schreiber, & Smoliga, 2017), serves as the primary approach.

Additionally, mental health professionals can offer guidance, develop structured strategies, and monitor progress, adjusting therapy as needed (Szabo & Demetrovics, 2022). However, because EA often occurs alongside other disorders, it is important to assess the effectiveness of psychotherapeutic interventions, as evidence for their success remains limited (Hadzlik et al., 2024; Weinstein & Szabo, 2023). While clinical trials for EA are indispensable, advancing treatment and prevention will ultimately depend on a more precise understanding of its etiology (Szabo, 2024). These treatment approaches discussed below have demonstrated effectiveness in related psychopathologies and may provide theoretically informed directions for future clinical evaluation in EA. Substantial empirical work is therefore needed to establish validated, disorder-specific interventions.

### Psychological interventions

#### Cognitive-behavioral therapy

Cognitive-behavioral therapy (CBT; Beck & Weishaar, 2000) in EA focuses on strengthening schemas linked to new, healthier information through consistent practice and reinforcement at each step. It emphasizes replacing old, irrational, or maladaptive schemas with adaptive ones, guiding the patient toward more appropriate actions (Szabo & Demetrovics, 2022). CBT is considered a preferred therapeutic approach (Olave et al., 2025) and aims not to eliminate exercise entirely but to help the patient identify addictive, maladaptive patterns and adopt a more balanced approach, understanding optimal levels, and focusing on exercise reprogramming (Hausenblas et al., 2017). CBT for behavioral addictions is the most well-supported treatment in terms of empirical evidence for its effectiveness (Brand et al., 2025). Recently, Szabo and Demetrovics (2022) proposed a CBT-based 10-step hierarchical approach, although its clinical utility has yet to be evaluated (Weinstein & Szabo, 2023).

#### Rational emotive behavior therapy

Rational emotive behavior therapy (REBT; Ellis, 1957) is a cognitive-behavioral approach designed to reduce irrational beliefs and reinforce the rational ones to enhance psychological well-being (Davis & Turner, 2020). This therapy posits that individuals' beliefs about themselves, others, and the world influence their emotional and behavioral responses to life's stressors (Ellis, 1957). A recent study by Knapp, Miller, Outar, and Turner (2023) demonstrated that REBT effectively reduces EA, associated irrational beliefs, and emotional distress. By re-evaluating exercise beliefs and goals, REBT could serve as an efficient treatment.

#### Cue exposure therapy

The goal of cue exposure therapy in EA treatment is to create mental dissociation (Szabo & Demetrovics, 2022). This is accomplished by repeatedly exposing the individual to cues under the guidance of a psychologist, without allowing them to engage in the conditioned exercise behavior, thereby disrupting the addiction cycle. Subsequently, cravings, urges, withdrawal symptoms, and self-concept are assessed and tied with coping strategies to help reduce painful cravings. Additionally, *cue replacement* (Cubillas, Vadillo, & Matute, 2017), a creative intervention, is also utilized when the individual is triggered by a cue with an urge to exercise. This behavior is paired with a new behavior, for instance, a social interaction or simply relaxation (and the new pairing is mentally strengthened until the goal is achieved) (Szabo & Demetrovics, 2022).

#### Systematic desensitization

Systematic desensitization (McGlynn, 2010) involves visualizing and accepting gradually lower levels of exercise while learning to control exercise behaviors. Successful reductions are reinforced through continuous consultation, which helps establish a realistic plan for decreasing exercise volume. Realistic goal setting is key, focusing on short-term objectives with personal rewards for achievements. Over time, individuals are expected to gain control and reduce their dependence on exercise, aiming for moderate and enjoyable physical activity rather than complete cessation (Szabo & Demetrovics, 2022).

#### Substitution therapy

Substitution therapy presents a valuable approach for addressing EA (Szabo & Demetrovics, 2022). It encourages individuals to explore and find pleasure in alternative activities that can be integrated into their daily routine, serving as a substitute for problematic exercise. These activities may range from engaging in cultural events, watching movies, meditation, yoga, and/or participating in meaningful volunteer work. The primary goal is to foster participation in socially and personally rewarding experiences, promoting a more holistic sense of well-being (Eisenberg et al., 1998). Ultimately, this approach aims to reduce exercise dependence, although individual treatment outcomes may be influenced by barriers, known as *treatment thresholds* (Stöver, 2011; Szabo & Demetrovics, 2022).

#### Acceptance and commitment therapy

Hayes, Strosahl, and Wilson (1999) developed acceptance and commitment therapy (ACT) which integrates mindfulness (Harris, 2006), acceptance, attention, and behavior change strategies to reduce psychological distress (El Rafihi-Ferreira et al., 2024). The main goal of ACT is to enhance psychological flexibility, enabling individuals to act adaptively (Lim, Voon, Yahya, Mohamad, & Ahmi, 2024; Paliliunas, Belisle, & Dixon, 2018) and create a sense of purpose in life through a mindful action (Anusuya & Gayatridevi, 2025) (aptly represented in ‘ACT’ as its abbreviation) (Harris, 2006). Although ACT incorporates elements of mindfulness, it goes beyond just mindfulness or meditation practices in its emphasis on behavioral change through values-based committed action rather than sustained meditative practice. ACT views formal mindfulness meditation as just one of several methods for developing mindfulness skills, which include acceptance, awareness of the present moment, cognitive defusion, and observing the self objectively (Harris, 2006). In contrast to mindfulness-based treatment, ACT is a more structured psychotherapy. It employs mindfulness and uses it as a way to promote psychological flexibility with value-consistent action rather than as a standalone meditative discipline.

***ACT in exercise addiction***. In an EA treatment context, Szabo and Demetrovics (2022) asserted that this therapy can be applied by helping addicted individuals face and embrace the cravings that trigger the impulsive need to engage in exercise behavior. The goal is to change maladaptive schemas by creating new, healthier schema associations. Here, individuals mindfully reconsider and accept the feelings associated with the fear of missing an exercise session. As these new schema connections develop, problematic exercise patterns are expected to gradually diminish and eventually cease. The core of ACT is the *change* in an individual's relationship with exercise, creating alternate adaptive schemas, psychological flexibility, and consequent behavior change that helps to reduce addictive patterns (Hayes, Luoma, Bond, Masuda, & Lillis, 2006; Szabo & Demetrovics, 2022).

#### Meditation and mindfulness

Over the past decade, mental training techniques such as spiritual meditation and mindfulness have emerged as effective treatments for various addictions, including both behavioral (Sancho et al., 2018) and substance-related addictions (Garland & Howard, 2018; Sancho et al., 2018). At their core, mindfulness-based treatment involves informal training, focusing primarily on attention-based meditation and present-moment awareness (Verhaeghen, 2021). Unlike ACT, which embeds mindfulness within a broader behavioral and cognitive framework (Anusuya & Gayatridevi, 2025; Hayes, Pistorello, & Levin, 2012), meditation and mindfulness interventions focus on cultivating sustained, non-judgmental awareness of the present experiences (Harris, 2006; Kabat-Zinn, 2003; Verhaeghen, 2021). To draw parallels, ACT, specifically targets cognitive fusion (becoming entangled in one's thoughts) and experiential avoidance (avoiding internal discomfort), whereas mindfulness interventions emphasize only non-judgmental observation of thoughts and emotions rather than a formal values-driven committed action.

***Mindfulness in substance-use disorders.*** A recent literature review by Krishnan (2024) highlighted the efficacy of yogic practices such as Sudarshan Kriya yoga and meditative breathwork as promising interventions for substance-use disorders (SUDs), particularly alcohol dependence. Similarly, Ray et al. (2024) underscored both the immediate and long-term benefits of mindfulness-guided meditation in supporting recovery from SUDs, specifically opioid use disorder. Garland and Howard (2018) echoed this by explaining that mindfulness meditation may be an effective approach for reducing substance misuse and cravings by regulating cognitive and psychophysiological processes critical to self-regulation and reward processing.

***Mindfulness in behavioral addictions.*** In alignment with this, Samanta et al. (2024) outlined that mindfulness-based interventions, as supported by prior research, can also play a significant role in managing behavioral addictions, decreasing internet and smartphone addiction, and serving as a promising treatment approach. Moreover, Tharumiya, Riniprabha, Sakthivel, Janani, and Manicka (2024) indicated that mindfulness significantly reduces gaming addiction, and Brunelle and Grossman (2022) suggested that it also mitigates compulsive buying behavior. Similarly, Melero Ventola, Yela, Crego, and Ortés-Rodríguez (2020), Shirk, Muquit, Deckro, Sweeney, and Kraus (2022), and van der Tempel (2019) all asserted that mindfulness-based interventions offer a compelling approach to reducing addictive symptoms of a behavioral addiction (such as gambling addiction).

***Neuroscientific evidence.*** Neurological research has highlighted that substance and behavioral addictions have similar anomalies in executive functioning including attention, planning, problem-solving, mood regulation, reward-sensitivity, and decreased sense of self-control (De Luca, Negri, & Bersani, 2023; Goldstein & Volkow, 2002). Therefore, a similar treatment application for EA may be helpful in this context. Mindfulness and yogic practices have been shown to reduce stress and anxiety effectively (Mohanty & Mitra, 2024; Monteiro, Galhardo, Senra, Pinto‐Gouveia, & Cunha, 2024; Tran et al., 2024), suggesting their potential as valuable coping strategies for EA. Finally, Anandkumar, Manivasagam, Kee, and Meyding-Lamade (2018) demonstrated that mindfulness has been successfully used alongside other treatments to address symptoms of EA, warranting further exploration in clinical settings (Szabo & Demetrovics, 2022). Concomitantly, Brand et al. (2025) also acknowledged the potential of mindfulness-based interventions in alleviating symptoms in behavioral addictions. However, they emphasized that the current evidence remains limited and heterogeneous.

### Pharmacological intervention

Pharmacological treatment for EA is scarce, primarily because it is not classified as a clinical disorder, which limits the availability of prescribed medications (Szabo & Demetrovics, 2022). Several treatments, including mood stabilizers, antidepressants, glutamatergic modulators, opioid antagonists, and antipsychotics, have demonstrated effectiveness in managing addictive symptoms and curbing cravings (De Luca et al., 2023; Marazziti, Presta, Baroni, Silvestri, & Dell’Osso, 2014). However, studies on pharmacological interventions for EA are lacking (De Luca et al., 2023).

#### Case-based evidence

One study offers a potentially promising trajectory for further investigation. Di Nicola et al. (2010) reported significant improvement in a 47-year-old male patient with bipolar type I disorder who also exhibited comorbid EA and compulsive buying behavior. Treatment with Quetiapine (an antipsychotic medication that interacts with dopamine D1 and D2, adrenergic α1 and α2, and serotonin 5-HT1A and 5HT2 receptors) yielded promising results. Although the medication was prescribed primarily to manage bipolar disorder and its comorbidities rather than specifically for EA, it also unexpectedly led to improvements in the reported compulsive behaviors (Szabo & Demetrovics, 2022). After four weeks of Quetiapine treatment, there was a slight reduction in compulsive buying and a moderate decrease in compulsive exercise. By 12 weeks, the symptoms had significantly diminished, and after 24 weeks, they had completely subsided. The patient's score on the EAI (Terry et al., 2004) dropped from 28 (‘at-risk’ category) upon admission to 12 (‘asymptomatic category’) following treatment (Di Nicola et al., 2010; Szabo & Demetrovics, 2022). Although this case highlights the potential of medications such as Quetiapine in addressing behavioral addictions, further research is needed to determine their efficacy (Di Nicola et al., 2010; Weinstein & Weinstein, 2014).

#### Neurotransmitter-based pharmacotherapy

Additionally, pharmacotherapy has shown success in managing many other behavioral addictions (De Luca et al., 2023). Beyond antipsychotics, selective serotonin reuptake inhibitors (SSRIs) play a crucial role because they influence the serotonergic system (De Luca et al., 2023). Studies suggest that SSRIs such as Citalopram (Zimmerman, Breen, & Posternak, 2002), Paroxetine (Kim, Grant, Adson, Shin, & Zaninelli, 2002), Escitalopram (Black, Shaw, Forbush, & Allen, 2007), and Fluvoxamine (Hollander et al., 2000) help alleviate compulsive behaviors and improve quality of life for those with a gambling addiction. Similar results have been observed in shopping addiction (McElroy, Satlin, Pope, Keck, & Hudson, 1991), and sex addiction (Stein et al., 1992).

Moreover, naltrexone, an opioid *μ*-receptor antagonist, is widely used in the treatment of alcohol and opioid dependence (De Luca et al., 2023). It has been effective in reducing compulsive pornography use, online addictions (Bostwick & Bucci, 2008), gambling addiction (Grant, Potenza, Hollander, & et al., 2006) and compulsive shopping (Bullock & Koran, 2003). Additionally, neurochemical evidence suggests that glutamate imbalance may worsen compulsive behaviors (Pittenger, 2015), while enhancing glutamatergic tone through glutamatergic modulators can help reduce addictive tendencies (Marazziti et al., 2014). N-acetylcysteine has demonstrated potential in treating gambling addiction (Grant, Kim, & Odlaug, 2007), while memantine may aid in controlling shopping addiction (Grant, Odlaug, Mooney, O’Brien, & Kim, 2012). Lastly, mood stabilizers such as lithium salts and topiramate monotherapy have proven effective in managing gambling addiction (Dannon, Lowengrub, Gonopolski, Musin, & Kotler, 2005; Pallanti, Quercioli, Sood, & Hollander, 2002).

While many different types of medications have been successful in treating behavioral addictions leading to guidelines that could also potentially be extended to EA, research continues to advance (De Luca et al., 2023). At the same time, it is important to note that unless EA is officially classified as a distinct mental dysfunction, pharmacological treatment is unlikely to be pursued (Szabo & Demetrovics, 2022). The first line of treatment for EA is rooted in psychotherapy (De Luca et al., 2023). Research has explored the effectiveness of both CBT and pharmacological intervention. However, their overall efficacy remains uncertain because they may target different underlying causes of EA (Weinstein & Szabo, 2023).

## Prevention

### Balanced exercise practices

Although the advantages of exercise are often celebrated, it is crucial to approach the benefits with caution because uncontrolled exercise can negatively impact an individual's health (Berczik et al., 2012; De Luca et al., 2023). Therefore, establishing a well-structured, balanced, and monitored fitness routine with planned rest periods is vital for allowing the body to recover effectively. Cultivating a positive environment that values athlete well-being over performance results could help mitigate the risk of maladaptive exercise behaviors (Vítková, Rusnáková, & Mudrák, 2025). Society plays a significant role in this process, and raising awareness of EA among sports stakeholders is crucial (Bulgay et al., 2025; Ceci et al., 2023).

### Awareness initiatives

In this regard, developing targeted educational programs in universities and sports organizations that promote intrinsic goals for optimal physical activity, such as enjoyment and holistic wellbeing, could help shift the focus away from external validation and appearance-based motives. Student wellness programs and university counselling services largely play a significant role in driving such initiatives, thereby preventing EA (Olave et al., 2025). Equally important is public education on evidence-based physical activity updated guidelines, including those outlined by the WHO, which specify optimal weekly exercise durations (in minutes) tailored to intensity levels. At the same time, psychoeducation is essential for the masses to alleviate appearance-related social pressures and motivate individuals to embrace their bodies. Fostering positive body image perceptions is vital, and policymakers can utilize these strategies to design public health campaigns that advocate for positive body image, balanced exercise habits, diminish appearance-related social pressures, and support the mindful use of fitness environments (Guo et al., 2025).

### Early detection and intervention

Given the highly individualized nature of addiction, addressing it effectively also necessitates a personalized response (Griffiths et al., 2023) and a tailored treatment approach (Godoy-Izquierdo, Navarrón, et al., 2023). Early detection of warning signs, such as engaging in physical activity despite injury or illness, experiencing guilt when unable to exercise, or a perceived loss of control over exercise behavior, is indispensable (Lichtenstein et al., 2021). Recognizing these signs early is essential, as timely intervention can ensure individuals receive appropriate support and treatment (Bulgay et al., 2025; De Luca et al., 2023). Furthermore, making such interventions accessible through remote platforms can help extend their reach, particularly during unforeseen circumstances that limit in-person services (Ceci et al., 2023). Therefore, identifying the risks posed by EA is important, but the solution cannot involve altogether ceasing physical activity. Instead, the focus should be on identifying and maintaining optimal, healthy levels of exercise that foster psychological, social, spiritual, and physical well-being—ultimately enhancing overall quality of life (Landolfi, 2013).

## The way forward

### Reconceptualizing exercise addiction

The present focused and evaluative narrative review encapsulates trends from the past decades of EA research, including its definition and symptomology, differentiation between primary and secondary EA, assessment issues, epidemiology, comorbidities, theoretical models adopted, and treatment interventions. Despite long-standing research efforts in EA, progress in the field remains modest. More than 1,000 publications have generated little clinically grounded knowledge. This imbalance highlights the need to systematically identify and address the field's core gaps (Szabo, 2024). A promising way forward would be to reconceptualize EA through clinical cases and derive insights inductively because the extensive research far outweighs the relatively few genuine cases that actually require clinical attention. Understanding the difference between addictive exercise and passionate exercise is also pivotal because the two types of excessive exercise share variance (Chhabra et al., 2024, 2025; Szabo, 2024). Controlling for known covariates of EA is therefore essential in future research. The field urgently needs a critical paradigm shift towards studying *exercise addiction* itself rather than its poorly defined *risk* construct (Chhabra et al., 2025). However, it is also imperative to strike a balance between not pathologizing routine behaviors, while also not overlooking conditions that demand genuine clinical attention and public health relevance (Brand et al., 2022).

### Strengthening clinical-research collaboration

Inspired by Szabo's (2001) pyramid approach, fostering the much-needed collaboration between clinicians and academic researchers is necessary. This can be done by conducting follow-up clinical (and qualitative) interviews of those viewed as at-risk of EA using screening instruments (Szabo et al., 2015). It is also crucial to develop a robust, and a highly specific tool through such deep interviews (Szabo, 2024). Additionally, studying clinical cases (Colledge, Buchner, Schmidt, & Walter, 2019) would also provide evidence for clinical recognition in future diagnostic frameworks (Szabo, 2024). Notably, a list of terms used for EA should be compiled to prevent exhaustive and redundant searches with synonymous meanings related to EA. Employing the correct screening tool is essential because EA often coexists with other psychiatric disorders (Colledge et al., 2019). More specifically, when studying team and individual sports, future research should evaluate ‘additional leisure-time activity’ among team-based participants to better identify EA (Griffiths et al., 2023).

### Research and policy directions

Larger sample studies are needed to enhance understanding of this subject (De Luca et al., 2023), and longitudinal (Gjoneska et al., 2024; Olave et al., 2025; Vítková et al., 2025), as well as experimental research is recommended (Godoy-Izquierdo, Ramírez, et al., 2023). Future studies would also benefit from employing cross-national study designs to deepen the understanding of how differing cultural contexts influence the assessment of behavioral addictions (Chhabra, Árok, & Szabo, 2024; Gjoneska et al., 2024). Finally, importance should be given to any necessary policies required for sustaining treatment approaches (Berczik et al., 2012). Limited guidance and research on EA can hinder diagnosis and treatment.

A key step forward is to ensure that the diagnosis of EA demonstrates clear harm, negative aspects, and a substantiated causal link to maladaptive exercise behavior (Szabo et al., 2015). Although not included in the DSM-5 or ICD-11, it is still important to inform healthcare professionals to detect any early signs of EA and prevent injuries or other detrimental effects on physiological and psychological health (De Luca et al., 2023). By synthesizing key insights into exercise addiction, the present narrative review provides a basis for its fundamental reappraisal and contributes to the advancement of the effective identification and management of this disorder.

## References

[B1] Abrantes, A. M., Farris, S. G., Brown, R. A., Greenberg, B. D., Strong, D. R., McLaughlin, N. C., & Riebe, D. (2019). Acute effects of aerobic exercise on negative affect and obsessions and compulsions in individuals with obsessive-compulsive disorder. *Journal of Affective Disorders*, 245, 991–997. 10.1016/j.jad.2018.11.074 https://doi.org/10.1016/j.jad.2018.11.074 30699885 PMC7037579

[B2] Ackard, D. M., Brehm, B. J., & Steffen, J. J. (2002). Exercise and eating disorders in college-aged women: Profiling excessive exercisers. *Eating Disorders: The Journal of Treatment & Prevention*, 10(1), 31–47. 10.1080/106402602753573540 https://doi.org/10.1080/106402602753573540 16864243

[B3] Ahorsu, D. K., Imani, V., Potenza, M. N., Chen, H. P., Lin, C. Y., & Pakpour, A. H. (2023). Mediating roles of psychological distress, insomnia, and body image concerns in the association between exercise addiction and eating disorders. *Psychology Research and Behavior Management*, 16, 2533–2542. 10.2147/PRBM.S414543 https://doi.org/10.2147/PRBM.S414543 37431433 PMC10329837

[B4] Akbari, M., Seydavi, M., Zamani, E., & Griffiths, M. D. (2024). The risk of exercise addiction mediates the relationship between social media use and mental health indices among young Iranians. *Addiction Research & Theory*, 32(1), 27–37. 10.1080/16066359.2022.2149742 https://doi.org/10.1080/16066359.2022.2149742

[B6] Alcaraz-Ibáñez, M., Paterna, A., Griffiths, M. D., & Demetrovics, Z. (2024a). Measurement invariance of the Exercise Addiction Inventory according to eating disorder risk status. *International Journal of Mental Health and Addiction*, 22(3), 1452–1462. 10.1007/s11469-022-00936-5 https://doi.org/10.1007/s11469-022-00936-5

[B7] Alcaraz-Ibanez, M., Paterna, A., Griffiths, M. D., Demetrovics, Z., & Sicilia, A. (2022b). Gender-related differences in self-reported problematic exercise symptoms: A systematic review and meta-analysis. *Psychology of Sport and Exercise*, 63, 102280. 10.1016/j.psychsport.2022.102280 https://doi.org/10.1016/j.psychsport.2022.102280

[B8] Alcaraz-Ibanez, M., Paterna, A., Griffiths, M. D., & Sicilia, A. (2024b). Psychometric properties of problematic exercise measures: A systematic review. *International Review of Sport and Exercise Psychology*, 17(2), 1013–1049. 10.1080/1750984X.2022.2111664 https://doi.org/10.1080/1750984X.2022.2111664

[B9] Alcaraz-Ibáñez, M., Paterna, A., Sicilia, Á., & Griffiths, M. D. (2020). Morbid exercise behaviour and eating disorders: A meta-analysis. *Journal of Behavioral Addictions*, 9(2), 206–224. 10.1556/2006.2020.00027 https://doi.org/10.1556/2006.2020.00027 32644935 PMC8939419

[B10] Alcaraz-Ibáñez, M., Paterna, A., Sicilia, Á., & Griffiths, M. D. (2021). A systematic review and meta-analysis on the relationship between body dissatisfaction and morbid exercise behaviour. *International Journal of Environmental Research and Public Health*, 18(2), 585. 10.3390/ijerph18020585 https://doi.org/10.3390/ijerph18020585 33445591 PMC7827926

[B11] Alcaraz-Ibáñez, M., Paterna, A., Sicilia, Á., & Griffiths, M. D. (2022a). Examining the reliability of the scores of self-report instruments assessing problematic exercise: A systematic review and meta-analysis. *Journal of Behavioral Addictions*, 11(2), 326–347. 10.1556/2006.2022.00014 https://doi.org/10.1556/2006.2022.00014 35482912 PMC9295230

[B12] American Psychiatric Association. (2022). *Diagnostic and statistical manual of mental disorders, 5th edition, text revision*. American Psychiatric Publishing.

[B13] Anandkumar, S., Manivasagam, M., Kee, V. T. S., & Meyding-Lamade, U. (2018). Effect of physical therapy management of nonspecific low back pain with exercise addiction behaviors: A case series. *Physiotherapy Theory and Practice*, 34(4), 316–328. 10.1080/09593985.2017.1394410 https://doi.org/10.1080/09593985.2017.1394410 29111859

[B14] Anusuya, S. P., & Gayatridevi, S. (2025). Acceptance and commitment therapy and psychological well-being: A narrative review. *Cureus*, 17(1), e77705. 10.7759/cureus.77705 https://doi.org/10.7759/cureus.77705 39974259 PMC11837766

[B15] Asadi Majareh, S., Moghtader, L., & Mousavi, S. M. (2021). The effectiveness of systematic desensitization and self-regulating on students' internet addiction. *Quarterly Journal of Child Mental Health*, 8(1), 97–109. 10.52547/jcmh.8.1.8 https://doi.org/10.52547/jcmh.8.1.8

[B16] Australian Institute of Health & Welfare. (2017). Impact of physical inactivity as a risk factor for chronic conditions: Australian burden of disease. Retrieved October 8, 2024, from https://www.aihw.gov.au/reports/burden-of-disease/impact-of-physical-inactivity-chronic-conditions/summary.

[B17] Baekeland, F. (1970). Exercise deprivation: Sleep and psychological reactions. *Archives of General Psychiatry*, 22(4), 365–369. 10.1001/archpsyc.1970.01740280077014 https://doi.org/10.1001/archpsyc.1970.01740280077014 4313770

[B18] Bamber, D. J., Cockerill, I. M., Rodgers, S., & Carroll, D. (2003). Diagnostic criteria for exercise dependence in women. *British Journal of Sports Medicine*, 37(5), 393–400. 10.1136/bjsm.37.5.393 https://doi.org/10.1136/bjsm.37.5.393 14514528 PMC1751359

[B19] Barker, J. L., Kolar, D., Lazzer, A. S. D., & Keel, P. K. (2022). Exercise satiation: A novel theoretical conceptualization for problematic exercise observed in eating disorders. *International Journal of Eating Disorders*, 55(2), 176–179. 10.1002/eat.23635 https://doi.org/10.1002/eat.23635 34729798

[B20] Beck, A. T., & Weishaar, M. E. (2000). Cognitive therapy. In R. J. Corsini, & D. Wedding (Eds.), *Current psychotherapies* (6th ed., pp. 241–272). Peacock.

[B21] Berczik, K., Szabó, A., Griffiths, M. D., Kurimay, T., Kun, B., Urbán, R., & Demetrovics, Z. (2012). Exercise addiction: Symptoms, diagnosis, epidemiology, and etiology. *Substance Use & Misuse*, 47(4), 403–417. 10.3109/10826084.2011.639120 https://doi.org/10.3109/10826084.2011.639120 22216780

[B22] Bircher, J., Griffiths, M. D., Kasos, K., Demetrovics, Z., & Szabo, A. (2017). Exercise addiction and personality: A two-decade systematic review of the empirical literature (1995-2015). *Baltic Journal of Sports and Health Sciences*, 3(106), 19–33. 10.33607/bjshs.v3i106.30 https://doi.org/10.33607/bjshs.v3i106.30

[B23] Black, D. W., Shaw, M., Forbush, K. T., & Allen, J. (2007). An open-label trial of escitalopram in the treatment of pathological gambling. *Clinical Neuropharmacology*, 30(4), 206–212. 10.1097/WNF.0b013e318032efcd https://doi.org/10.1097/WNF.0b013e318032efcd 17762317

[bib232] Blumenthal, J. A., O’Toole, L. C., & Chang, J. L. (1984). Is running an analogue of anorexia nervosa? An empirical study of obligatory running and anorexia nervosa. *JAMA*, 252(4), 520–523. 10.1001/jama.1984.03350040050022 http://doi.org/10.1001/jama.1984.03350040050022 6737645

[B24] Boone, T. (1990). Obsessive exercise – some reflections. *Journal of Physical Education, Recreation & Dance*, 61(7), 45–49. 10.1080/07303084.1990.10604579 https://doi.org/10.1080/07303084.1990.10604579

[B25] Bostwick, J. M., & Bucci, A. (2008). Internet sex addiction treated with naltrexone. *Mayo Clinic Proceedings*, 83(2), 226–230. 10.4065/83.2.226 https://doi.org/10.4065/83.2.226 18241634

[B26] Brand, M., Antons, S., Bőthe, B., Demetrovics, Z., Fineberg, N. A., Jimenez-Murcia, S., … Potenza, M. N. (2025). Current advances in behavioral addictions: From fundamental research to clinical practice. *American Journal of Psychiatry*, 182(2), 155–163. 10.1176/appi.ajp.20240092 https://doi.org/10.1176/appi.ajp.20240092 39659159

[B27] Brand, M., Rumpf, H., Demetrovics, Z., Müller, A., Stark, R., King, D. L., … Potenza, M. N. (2022). Which conditions should be considered as disorders in the International Classification of Diseases (ICD-11) designation of “other specified disorders due to addictive behaviors”. *Journal of Behavioral Addictions*, 11(2), 150–159. 10.1556/2006.2020.00035 https://doi.org/10.1556/2006.2020.00035 32634114 PMC9295220

[bib208] Brown, R. I. F. (1997). A theoretical model of the behavioral addictions: Applied to offending. In Hodge, J. E., McMurran, M., & Hollin, C. R. (Eds.), *Addicted to crime?* (pp. 15–63). Wiley.

[B28] Brunelle, C., & Grossman, H. (2022). Predictors of online compulsive buying: The role of personality and mindfulness. *Personality and Individual Differences*, 185, 111237. 10.1016/j.paid.2021.111237 https://doi.org/10.1016/j.paid.2021.111237

[B29] Bueno-Antequera, J., Legaz-Arrese, A., Paris-Garcia, F., Guille, R., Munguı, D., & Mayolas-Pi, C. (2022). Exercise addiction stability and health effects. A 6-month follow-up postcompetition study in amateur endurance cyclists. *Journal of Addiction Medicine*, 16(3), e140–e149. 10.1097/ADM.0000000000000897 https://doi.org/10.1097/ADM.0000000000000897 34145189

[B30] Bueno-Antequera, J., Mayolas-Pi, C., Reverter-Masià, J., López-Laval, I., Oviedo-Caro, M. Á., Munguía-Izquierdo, D., … Legaz-Arrese, A. (2020). Exercise addiction and its relationship with health outcomes in indoor cycling practitioners in fitness centers. *International Journal of Environmental Research and Public Health*, 17(11), 4159. 10.3390/ijerph17114159 https://doi.org/10.3390/ijerph17114159 32545197 PMC7312881

[B31] Bulgay, C., Kasakolu, A., Bıyıklı, T., Koncagul, S., Kazan, H. H., Ahmetov, I. I., … Szabo, A. (2025). Genome-wide association study of exercise addiction among elite wrestlers. *Brain Sciences*, 15(2), 102. 10.3390/brainsci15020102 https://doi.org/10.3390/brainsci15020102 40002435 PMC11853435

[B32] Bullock, K., & Koran, L. (2003). Psychopharmacology of compulsive buying. *Drugs Today*, 39(9), 695–700. 10.1358/dot.2003.39.9.796664 https://doi.org/10.1358/dot.2003.39.9.796664 14586484

[B33] Çakın, G., Juwono, I. D., Potenza, M. N., & Szabo, A. (2021). Exercise addiction and perfectionism: A systematic review of the literature. *Current Addiction Reports*, 8, 144–155. 10.1007/s40429-021-00358-8 https://doi.org/10.1007/s40429-021-00358-8

[B34] Calogero, R. M., & Pedrotty, K. N. (2004). The practice and process of healthy exercise: An investigation of the treatment of exercise abuse in women with eating disorders. *Eating Disorders*, 12(4), 273–291. 10.1080/10640260490521352 https://doi.org/10.1080/10640260490521352 16864521

[B35] Caspersen, C. J., Powell, K. E., & Christenson, G. M. (1985). Physical activity, exercise, and physical fitness: Definitions and distinctions for health-related research. *Public health Reports*, 100(2), 126. 3920711 PMC1424733

[B36] Ceci, F., Di Carlo, F., Burkauskas, J., Salone, A., De Luca, I., Cicconcelli, D., … Corazza, O. (2023). Physical activity and exercise addiction during the Covid-19 pandemic in Italy. *International Journal of Mental Health and Addiction*, 21(6), 3678–3698. 10.1007/s11469-022-00815-z https://doi.org/10.1007/s11469-022-00815-z PMC9020546 35469185

[B37] Čekić, D. (2024). Psychology of exercise as a foundation for determining the benefits of exercise for the mental health of the modern individual. *Sport Media and Business*, 10(2), 73–86. 10.58984/smb2402073c https://doi.org/10.58984/smb2402073c

[bib224] Chapman, C. L., & De Castro, J. M. (1990). Running addiction: Measurement and associated psychological characteristics. *Journal of Sports Medicine and Physical Fitness*, 30(3), 283–290. PMID: 2266760. 2266760

[B38] Chhabra, B., Árok, P., & Szabo, A. (2024c). A cross-cultural examination of passion and risk of exercise addiction in Hungarian and Indian exercisers. *International Journal of Sport and Exercise Psychology*, Online first, 1–18. 10.1080/1612197X.2024.2446324 https://doi.org/10.1080/1612197X.2024.2446324

[B39] Chhabra, B., Bartos, A., Soós, I., Ruíz‐Barquín, R., de la Vega, R., & Szabo, A. (2025). Passion, perfectionism, and sports commitment as predictors of exercise addiction. *European Journal of Sport Science*, 25(11), e70068. 10.1002/ejsc.70068 https://doi.org/10.1002/ejsc.70068 41108529 PMC12535194

[B40] Chhabra, B., Granziol, U., Griffiths, M. D., Zandonai, T., Landolfi, E., Solmi, M., … Szabo, A. (2024b). Prevalence of the risk of exercise addiction based on a new classification: A cross-sectional study in 15 countries. *International Journal of Mental Health and Addiction*, 23, 3815–3836. 10.1007/s11469-024-01322-z https://doi.org/10.1007/s11469-024-01322-z

[B41] Chhabra, B., Nazlıgül, M. D., & Szabo, A. (2024a). Exercise addiction in team sports: A systematic literature review. *Scandinavian Journal of Psychology*, 65(5), 846–857. 10.1111/sjop.13026 https://doi.org/10.1111/sjop.13026 38760321

[B42] Collado-Boira, E., Temprado-Albalat, M. D., Martínez-Navarro, I., Gandhi-Morar, K., Hernando-Fuster, B., Bernalte-Martí, V., & Hernando-Domingo, C. (2021). Variables related to exercise dependence and quality of life in amateur long-distance runners. *Medicina dello Sport*, 74(2), 295–312. 10.23736/S0025-7826.21.03771-6 https://doi.org/10.23736/S0025-7826.21.03771-6

[B43] Colledge, F., Buchner, U., Schmidt, A., & Walter, M. (2019). Does exercise addiction exist? A brief review on current measurement tools and future directions. *Mental Health and Addiction Research*, 4(2), 1–4. 10.15761/MHAR.1000181 https://doi.org/10.15761/MHAR.1000181

[B44] Colledge, F., Cody, R., Buchner, U. G., Schmidt, A., Pühse, U., Gerber, M., … Walter, M. (2020b). Excessive exercise—a meta-review. *Frontiers in Psychiatry*, 11, 521572. 10.3389/fpsyt.2020.521572 https://doi.org/10.3389/fpsyt.2020.521572 33329076 PMC7714788

[B45] Colledge, F., Sattler, I., Schilling, H., Gerber, M., Pühse, U., & Walter, M. (2020a). Mental disorders in individuals at risk for exercise addiction–A systematic review. *Addictive Behaviors Reports*, 12, 100314. 10.1016/j.abrep.2020.100314 https://doi.org/10.1016/j.abrep.2020.100314 33364322 PMC7752715

[B46] Coniglio, K. A., Davis, L., Sun, J., Loureiro, N., & Selby, E. A. (2023). Detecting pathological exercise in college men: An investigation using latent profile analysis. *Journal of American College Health*, 71(7), 2258–2262. 10.1080/07448481.2021.1965612 https://doi.org/10.1080/07448481.2021.1965612 34415230

[B47] Conn, K., Huang, K., Gorrell, S., & Foldi, C. J. (2024). A transdiagnostic and translational framework for delineating the neuronal mechanisms of compulsive exercise in anorexia nervosa. *International Journal of Eating Disorders*, 57(7), 1406–1417. 10.1002/eat.24130 https://doi.org/10.1002/eat.24130 38174745 PMC11222308

[B48] Cook, B., Hausenblas, H., & Freimuth, M. (2014). Exercise addiction and compulsive exercising: Relationship to eating disorders, substance use disorders, and addictive disorders. In T. Brewerton, & A. Baker Dennis (Eds.), *Eating disorders, addictions and substance use disorders: Research, clinical and treatment perspectives* (pp. 127–144). Springer. 10.1007/978-3-642-45378-6_7 https://doi.org/10.1007/978-3-642-45378-6_7

[B49] Cook, B., & Luke, R. (2017). Primary and secondary exercise dependence in a sample of cyclists. *International Journal of Mental Health and Addiction*, 15(2), 444–451. 10.1007/s11469-017-9745-z https://doi.org/10.1007/s11469-017-9745-z

[bib226] Corbin, C. B., Nielsen, A. B., Borsdorf, L. L., & Laurie, D. R. (1987). Commitment to physical activity. *International Journal of Sport Psychology*, 18, 215–222.

[B50] Cosh, S. M., Eshkevari, E., McNeil, D. G., & Tully, P. J. (2023). Classifying excessive exercise: Examining the relationship between compulsive exercise with obsessive‐compulsive disorder symptoms and disordered eating symptoms. *European Eating Disorders Review*, 31(6), 769–780. 10.1002/erv.3002 https://doi.org/10.1002/erv.3002 37353901

[B51] Cousineau, D., Ferguson, R. J., de Champlain, J., Gauthier, P., Cote, P., & Bourassa, M. (1977). Catecholamines in coronary sinus during exercise in man before and after training. *Journal of Applied Physiology*, 43(5), 801–806. 10.1152/jappl.1977.43.5.801 https://doi.org/10.1152/jappl.1977.43.5.801 591472

[B52] Cubillas, C. P., Vadillo, M. A., & Matute, H. (2017). Changes in cue configuration reduce the impact of interfering information in a predictive learning task. *Frontiers in Psychology*, 7, 2050. 10.3389/fpsyg.2016.02050 https://doi.org/10.3389/fpsyg.2016.02050 28111562 PMC5216052

[B53] Cunningham, H. E., Pearman III, S., & Brewerton, T. D. (2016). Conceptualizing primary and secondary pathological exercise using available measures of excessive exercise. *International Journal of Eating Disorders*, 49(8), 778–792. 10.1002/eat.22551 https://doi.org/10.1002/eat.22551 27203379

[B54] Dannon, P. N., Lowengrub, K., Gonopolski, Y., Musin, E., & Kotler, M. (2005). Topiramate versus fluvoxamine in the treatment of pathological gambling: A randomized, blind-rater, comparison study. *Clinical Neuropharmacology*, 28(1), 6–10. 10.1097/00002826-200501000-00002 https://doi.org/10.1097/00002826-200501000-00002 15711432

[B55] Davis, C. (2000). Exercise abuse. *International Journal of Sport Psychology*, 31(2), 278–289.

[bib211] Davis, C., Brewer, H., & Ratusny, D. (1993). Behavioral frequency and psychological commitment: Necessary concepts in the study of excessive exercising. *Journal of Behavioral Medicine*, 16(6), 611–628. 10.1007/BF00844722 https://doi.org/10.1007/BF00844722 8126715

[B56] Davis, H., & Turner, M. J. (2020). The use of rational emotive behavior therapy (REBT) to increase the self-determined motivation and psychological well-being of triathletes. *Sport, Exercise, and Performance Psychology*, 9(4), 489–505. 10.1037/spy0000191 https://doi.org/10.1037/spy0000191

[B57] De Luca, I., Negri, A., & Bersani, G. (2023). How to treat exercise addiction: Psychological interventions and new pharmacological perspectives. In O. Corazza, & A. Rocha Dores (Eds.), *The body in the mind: Exercise addiction, body image and the use of enhancement drugs* (Vol. 145, pp. 145–161). Cambridge University Press.

[bib214] DeBate, R. D., Huberty, J., & Pettee, K. (2009). Psychometric properties of the commitment to physical activity scale. *American Journal of Health Behavior*, 33(4), 425–434. 10.5993/AJHB.33.4.8 https://doi.org/10.5993/AJHB.33.4.8 19182987

[B58] Demir, G. T., Hazar, Z., & Cicioğlu, H. İ. (2018). Exercise Addiction Scale (EAS): A study of validity and reliability. *Kastamonu Education Journal*, 26(3), 865–874.

[B59] Di Lodovico, L., Poulnais, S., & Gorwood, P. (2019). Which sports are more at risk of physical exercise addiction: A systematic review. *Addictive Behaviors*, 93, 257–262. 10.1016/j.addbeh.2018.12.030 https://doi.org/10.1016/j.addbeh.2018.12.030 30595420

[B60] Di Nicola, M., Martinotti, G., Mazza, M., Tedeschi, D., Pozzi, G., & Janiri, L. (2010). Quetiapine as add-on treatment for bipolar I disorder with comorbid compulsive buying and physical exercise addiction. *Progress in Neuro-Psychopharmacology and Biological Psychiatry*, 34(4), 713–714. 10.1016/j.pnpbp.2010.03.013 https://doi.org/10.1016/j.pnpbp.2010.03.013 20298734

[B61] Dinardi, J. S., Egorov, A. Y., & Szabo, A. (2021). The expanded interactional model of exercise addiction. *Journal of Behavioral Addictions*, 10(3), 626–631. 10.1556/2006.2021.00061 https://doi.org/10.1556/2006.2021.00061 34524973 PMC8997218

[B62] Dishman, R. K., & O/Connor, P. J. (2009). Lessons in exercise neurobiology: The case of endorphins. *Mental Health and Physical Activity*, 2(1), 4–9. 10.1016/j.mhpa.2009.01.002 https://doi.org/10.1016/j.mhpa.2009.01.002

[B63] Dores, A. R., Carvalho, I. P., Burkauskas, J., Simonato, P., De Luca, I., Mooney, R., … Corazza, O. (2021). Exercise and use of enhancement drugs at the time of the COVID-19 pandemic: A multicultural study on coping strategies during self-isolation and related risks. *Frontiers in Psychiatry*, 12, 648501. 10.3389/fpsyt.2021.648501 https://doi.org/10.3389/fpsyt.2021.648501 33776822 PMC7988429

[bib215] Downs, D. S., Hausenblas, H. A., & Nigg, C. R. (2004). Factorial validity and psychometric examination of the exercise dependence scale-revised. *Measurement in Physical Education and Exercise Science*, 8(4), 183–201. 10.1207/s15327841mpee0804_1 https://doi.org/10.1207/s15327841mpee0804_1

[B64] Dumitru, D. C., Dumitru, T., & Maher, A. J. (2018). A systematic review of exercise addiction: Examining gender differences. *Journal of Physical Education and Sport*, 18(3), 1738–1747. 10.7752/jpes.2018.03253 https://doi.org/10.7752/jpes.2018.03253

[bib212] Duncan, L. R., Hall, C. R., Fraser, S. N., Rodgers, W. M., Wilson, P. M., & Loitz, C. C. (2012). Re-examining the dimensions of obligatory exercise. *Measurement in Physical Education and Exercise Science*, 16(1), 1–22. 10.1080/1091367X.2012.641442 https://doi.org/10.1080/1091367X.2012.641442

[B65] Egorov, A. Y., & Szabo, A. (2013). The exercise paradox: An interactional model for a clearer conceptualization of exercise addiction. *Journal of Behavioral Addictions*, 2(4), 199–208. 10.1556/jba.2.2013.4.2 https://doi.org/10.1556/jba.2.2013.4.2 25215201 PMC4154576

[B66] Eisenberg, D. M., Davis, R. B., Ettner, S. L., Appel, S., Wilkey, S., Van Rompay, M., & Kessler, R. C. (1998). Trends in alternative medicine use in the United States, 1990–1997: Results of a follow-up national survey. *JAMA*, 280(18), 1569–1575. 10.1001/jama.280.18.1569 https://doi.org/10.1001/jama.280.18.1569 9820257

[B67] El Rafihi-Ferreira, R., Hasan, R., Toscanini, A. C., Linares, I. M. P., Suzuki Borges, D., Brasil, I. P., … Morin, C. (2024). Acceptance and commitment therapy versus cognitive behavioral therapy for insomnia: A randomized controlled trial. *Journal of Consulting and Clinical Psychology*, 92(6), 330–343. 10.1037/ccp0000881 https://doi.org/10.1037/ccp0000881 39023982

[B68] Ellis, A. (1957). Rational psychotherapy and individual psychology. *Journal of Individual Psychology*, 13(1), 38–44. 10.1353/jip.2017.0023 https://doi.org/10.1353/jip.2017.0023

[B69] Ertl, M. M., Pazienza, R., Cannon, M., Cabrera Tineo, Y. A., Fresquez, C. L., McDonough, A. K., … Martin, J. L. (2022). Associations between impulsivity and exercise addiction, disordered eating, and alcohol use behaviors: A latent profile analysis. *Substance Use & Misuse*, 57(6), 886–896. 10.1080/10826084.2022.2052095 https://doi.org/10.1080/10826084.2022.2052095 35321617 PMC9019863

[B70] Fernandez-del-Valle, M., Quesnel, D. A., Mitchell, J. J., & Robert-McComb, J. J. (2023). Screening for eating disorders, dysfunctional exercise, and menstrual dysfunction in female athletes. In J. J. Robert-McComb, M. Zumwalt, & M. Fernandez-del-Valle (Eds.), *The active female: Health issues throughout the lifespan* (pp. 183–210). Springer International Publishing. 10.1007/978-3-031-15485-0_12 https://doi.org/10.1007/978-3-031-15485-0_12

[B71] Foster, A., Shorter, G., & Griffiths, M. (2015). Muscle dysmorphia: Could it be classified as an addiction to body image? *Journal of Behavioral Addictions*, 4(1), 1–5. 10.1556/jba.3.2014.001 https://doi.org/10.1556/jba.3.2014.001 PMC4394845 25592218

[B72] Freimuth, M., Moniz, S., & Kim, S. R. (2011). Clarifying exercise addiction: Differential diagnosis, co-occurring disorders, and phases of addiction. *International Journal of Environmental Research and Public Health*, 8(10), 4069–4081. 10.3390/ijerph8104069 https://doi.org/10.3390/ijerph8104069 22073029 PMC3210598

[B73] Furley, P., & Goldschmied, N. (2021). Systematic vs. narrative reviews in sport and exercise psychology: Is either approach superior to the other? *Frontiers in Psychology*, 12, 685082. 10.3389/fpsyg.2021.685082 https://doi.org/10.3389/fpsyg.2021.685082 34305741 PMC8299000

[B74] Ganson, K. T., Lavender, J. M., Rodgers, R. F., Cunningham, M., & Nagata, J. M. (2022). Compulsive exercise and vaping among a sample of US college students aged 18–26 years. *Eating and Weight Disorders-Studies on Anorexia, Bulimia and Obesity*, 27(3), 1153–1161. 10.1007/s40519-021-01251-z https://doi.org/10.1007/s40519-021-01251-z 34181209

[B75] Garland, E. L., & Howard, M. O. (2018). Mindfulness-based treatment of addiction: Current state of the field and envisioning the next wave of research. *Addiction Science & Clinical Practice*, 13, 14. 10.1186/s13722-018-0115-3 https://doi.org/10.1186/s13722-018-0115-3 29669599 PMC5907295

[bib216] Garman, J. F., Hayduk, D. M., Crider, D. A., & Hodel, M. M. (2004). Occurrence of exercise dependence in a college-aged population. *Journal of American College Health*, 52(5), 221–228. PMID: 15029944. 15029944 10.3200/JACH.52.5.221-228

[B76] Gillman, A. S., Hutchison, K. E., & Bryan, A. D. (2015). Cannabis and exercise science: A commentary on existing studies and suggestions for future directions. *Sports Medicine*, 45, 1357–1363. 10.1007/s40279-015-0362-3 https://doi.org/10.1007/s40279-015-0362-3 26178329

[B77] Gjoneska, B., Bőthe, B., Potenza, M. N., Szabo, A., & Demetrovics, Z. (2024). *Epidemiology of behavioral addictions* (pp. 47–59). The sage Handbook of addiction psychology.

[B78] Glasser, W. (1976). *Positive addiction*. Harper & Row.

[B79] Godoy-Izquierdo, D., Navarrón, E., López-Mora, C., & González-Hernández, J. (2023b). Exercise addiction in the sports context: What is known and what is yet to be known. *International Journal of Mental Health and Addiction*, 21(2), 1057–1074. 10.1007/s11469-021-00641-9 https://doi.org/10.1007/s11469-021-00641-9

[B80] Godoy-Izquierdo, D., Ramírez, M. J., Díaz, I., & López-Mora, C. (2023a). A systematic review on exercise addiction and the disordered eating-eating disorders continuum in the competitive sport context. *International Journal of Mental Health and Addiction*, 21(1), 529–561. 10.1007/s11469-021-00610-2 https://doi.org/10.1007/s11469-021-00610-2

[B81] Goldberg, A. (1988). The sports mind: A workbook of mental skills for athletes. *Competitive Advantage*.

[B82] Goldstein, R. Z., & Volkow, N. D. (2002). Drug addiction and its underlying neurobiological basis: Neuroimaging evidence for the involvement of the frontal cortex. *American Journal of Psychiatry*, 159(10), 1642–1652. 10.1176/appi.ajp.159.10.1642 https://doi.org/10.1176/appi.ajp.159.10.1642 12359667 PMC1201373

[B83] Gori, A., Topino, E., & Griffiths, M. D. (2024). Family functioning styles and exercise addiction: Disengaged, enmeshed, and rigid family patterns are associated with exercise addiction. *European Journal of Investigation in Health, Psychology and Education*, 14(1), 148–163. 10.3390/ejihpe14010010 https://doi.org/10.3390/ejihpe14010010 38248130 PMC10814248

[B84] Grant, J. E., Kim, S. W., & Odlaug, B. L. (2007). N-acetyl cysteine, a glutamate modulating agent, in the treatment of pathological gambling: A pilot study. *Biological Psychiatry*, 62(6), 652–657. 10.1016/j.biopsych.2006.11.021 https://doi.org/10.1016/j.biopsych.2006.11.021 17445781

[B85] Grant, J. E., Odlaug, B. L., Mooney, M., O’Brien, R., & Kim, S. W. (2012). Open-label pilot study of memantine in the treatment of compulsive buying. *Annals of Clinical Psychiatry*, 24(2), 119–126. 10.1177/104012371202400202 https://doi.org/10.1177/104012371202400202 22563566

[B86] Grant, J. E., Potenza, M. N., Hollander, E., Cunningham-Williams, R., Nurminen, T., Smits, G., & Kallio, A. (2006). Multicenter investigation of the opioid antagonist nalmefene in the treatment of pathological gambling. *American Journal of Psychiatry*, 163(2), 303–312. 10.1176/appi.ajp.163.2.303 https://doi.org/10.1176/appi.ajp.163.2.303 16449486

[B87] Granziol, U., Griffiths, M. D., Zou, L., Yang, P., Herschel, H. K., Junker, A., … Szabo, A. (2024). The Expanded Exercise Addiction Inventory (EAI-3): Towards reliable and international screening of exercise-related dysfunction. *International Journal of Mental Health and Addiction*, 22, 3559–3585. 10.1007/s11469-023-01066-2 https://doi.org/10.1007/s11469-023-01066-2 PMC10171173 37363769

[B88] Griffiths, M. D. (1996). Behavioural addictions: An issue for everybody? *Journal of Workplace Learning*, 8(3), 19–25. 10.1108/13665629610116872 https://doi.org/10.1108/13665629610116872

[B89] Griffiths, M. D. (2005). A “components” model of addiction within a biopsychosocial framework. *Journal of Substance Use*, 10(4), 191–197. 10.1080/14659890500114359 https://doi.org/10.1080/14659890500114359

[B90] Griffiths, M. D., Landolfi, E., & Szabo, A. (2023). Does exercise addiction exist among individuals engaged in team-based exercise? A position paper. *International Journal of Mental Health and Addiction*, 22, 3133–3148. 10.1007/s11469-023-01039-5 https://doi.org/10.1007/s11469-023-01039-5

[B91] Griffiths, M. D., Urbán, R., Demetrovics, Z., Lichtenstein, M. B., de la Vega, R., Kun, B., … Szabo, A. (2015). A cross-cultural re-evaluation of the Exercise Addiction Inventory (EAI) in five countries. *Sports Medicine Open*, 1, 1–7. 10.1186/s40798-014-0005-5 27747842 PMC4532705

[B92] Gunnarsson, B., Entezarjou, A., Fernández-Aranda, F., Jiménez-Murcia, S., Kenttä, G., & Håkansson, A. (2022). Understanding exercise addiction, psychiatric characteristics and use of anabolic androgenic steroids among recreational athletes – An online survey study. *Frontiers in Sports and Active Living*, 4, 903777. 10.3389/fspor.2022.903777 https://doi.org/10.3389/fspor.2022.903777 35979064 PMC9376369

[B93] Guo, S., Izydorczyk, B., Lipowska, M., Lizinczyk, S., Kamionka, A., Sajewicz-Radtke, U., … Lipowski, M. (2023). Sociocultural predictors of obligatory exercise in young men: A Polish-Chinese comparison. *Frontiers in Psychiatry*, 14, 1123864. 10.3389/fpsyt.2023.1123864 https://doi.org/10.3389/fpsyt.2023.1123864 37124264 PMC10130428

[B94] Guo, S., Kamionka, A., Xue, Q., Izydorczyk, B., Lipowska, M., & Lipowski, M. (2025). Body image and risk of exercise addiction in adults: A systematic review and meta-analysis. *Journal of Behavioral Addictions*, 14(1), 39–54. 10.1556/2006.2024.00085 https://doi.org/10.1556/2006.2024.00085 39912824 PMC11974424

[B95] Hądzlik, I., Wojtyła, K., & Barg, M. (2024). When healthy habits turn harmful: A medical perspective on exercise addiction. *Quality in Sport*, 20, 54154. 10.12775/QS.2024.20.54154 https://doi.org/10.12775/QS.2024.20.54154

[B96] Hailey, B. J., & Bailey, L. A. (1982). Negative addiction in runners: A quantitative approach. *Journal of Sport Behavior*, 5(3), 150–154.

[B97] Harris, R. (2006). Embracing your demons: An overview of acceptance and commitment therapy. *Psychotherapy in Australia*, 12(4), 70–76. 10.3316/informit.545561433272993 https://doi.org/10.3316/informit.545561433272993

[B98] Harris, A., Hay, P., & Touyz, S. (2020). Psychometric properties of instruments assessing exercise in patients with eating disorders: A systematic review. *Journal of Eating Disorders*, 8, 45. 10.1186/s40337-020-00315-2 https://doi.org/10.1186/s40337-020-00315-2 32884810 PMC7465430

[B208] Harris, A., Mannan, H., Hay, P., Aouad, P., Arcelus, J., Attia, E., Crosby, R., Madden, S., Meyer, C., & Touyz, S. (2024). Assessment and treatment of compulsive exercise in anorexia nervosa – A combined investigation of compulsive exercise activity therapy (LEAP) and compulsive exercise test subscales. *Eating Behaviors*, 52, 101825. 10.1016/j.eatbeh.2023.101825 https://doi.org/10.1016/j.eatbeh.2023.101825 38006774

[bib217] Hausenblas, H. A., & Downs, D. S. (2002). How much is too much? The development and validation of the exercise dependence scale. *Psychology & Health*, 17(4), 387–404. 10.1080/0887044022000004894 https://doi.org/10.1080/0887044022000004894

[B100] Hausenblas, H. A., Schreiber, K., & Smoliga, J. M. (2017). Addiction to exercise. *BMJ*, 357, j1745. 10.1136/bmj.j1745 https://doi.org/10.1136/bmj.j1745 28446435

[B101] Hayes, S. C., Luoma, J. B., Bond, F. W., Masuda, A., & Lillis, J. (2006). Acceptance and commitment therapy: Model, processes and outcomes. *Behaviour Research and Therapy*, 44(1), 1–25. 10.1016/j.brat.2005.06.006 https://doi.org/10.1016/j.brat.2005.06.006 16300724

[B102] Hayes, S. C., Pistorello, J., & Levin, M. E. (2012). Acceptance and commitment therapy as a unified model of behavior change. *The Counseling Psychologist*, 40(7), 976–1002. 10.1177/0011000012460836 https://doi.org/10.1177/0011000012460836

[B103] Hayes, S. C., Strosahl, K. D., & Wilson, K. G. (1999). *Acceptance and commitment therapy: An experiential approach to behavior change*. Guilford Press.

[B104] Hoekstra, R., Vugteveen, J., Warrens, M. J., & Kruyen, P. M. (2019). An empirical analysis of alleged misunderstandings of coefficient alpha. *International Journal of Social Research Methodology*, 22(4), 351–364. 10.1080/13645579.2018.1547523 https://doi.org/10.1080/13645579.2018.1547523

[B105] Hollander, E., DeCaria, C. M., Finkell, J. N., Begaz, T., Wong, C. M., & Cartwright, C. (2000). A randomized double-blind fluvoxamine/placebo crossover trial in pathologic gambling. *Biological Psychiatry*, 47(9), 813–817. 10.1016/S0006-3223(99)00255-2 https://doi.org/10.1016/S0006-3223(99)00255-2 10812040

[B107] Huang, W. Y., Huang, H., & Wu, C. E. (2022). Physical activity and social support to promote a health-promoting lifestyle in older adults: An intervention study. *International Journal of Environmental Research and Public Health*, 19(21), 14382. 10.3390/ijerph192114382 https://doi.org/10.3390/ijerph192114382 36361256 PMC9658453

[B108] Juwono, I. D., & Szabo, A. (2021). 100 cases of exercise addiction: More evidence for a widely researched but rarely identified dysfunction. *International Journal of Mental Health and Addiction*, 19, 1799–1811. 10.1007/s11469-020-00264-6 https://doi.org/10.1007/s11469-020-00264-6

[B109] Kabat-Kahn, R. (1972). *The boys of summer*. Harper & Row.

[B110] Kalayasiri, R., & Rattanawijarn, C. (2025). Demographics and physical and mental health of clients at a sports center with and without exercise addiction. *PeerJ*, 13, e19002. 10.7717/peerj.19002 https://doi.org/10.7717/peerj.19002 40151458 PMC11949105

[B111] Katzmarzyk, P. T., Friedenreich, C., Shiroma, E. J., & Lee, I. M. (2022). Physical inactivity and non-communicable disease burden in low-income, middle-income and high-income countries. *British Journal of Sports Medicine*, 56(2), 101–106. 10.1136/bjsports-2020-103640 https://doi.org/10.1136/bjsports-2020-103640 33782046 PMC8478970

[B112] Khoshro, S., & Abbasalizad Farhangi, M. (2024). Major dietary patterns, exercise addiction, and eating disorders among a sample of physically active young adults. *Nutrition and Metabolic Insights*, 17, 11786388241258938. 10.1177/11786388241258938 https://doi.org/10.1177/11786388241258938 39070982 PMC11273586

[B114] Kim, S. W., Grant, J. E., Adson, D. E., Shin, Y. C., & Zaninelli, R. (2002). A double-blind placebo-controlled study of the efficacy and safety of paroxetine in the treatment of pathological gambling. *Journal of Clinical Psychiatry*, 63(6), 501–507. 10.4088/JCP.v63n0609 https://doi.org/10.4088/JCP.v63n0609 12088161

[B115] Kline, T. J. B., Franken, R. E., & Rowland, G. L. (1994). A psychometric evaluation of the Exercise Salience Scale. *Personality and Individual Differences*, 16(3), 509–511. 10.1016/0191-8869(94)90078-7 https://doi.org/10.1016/0191-8869(94)90078-7

[B116] Knapp, S., Miller, A., Outar, L., & Turner, M. (2023). Psychological well-being and exercise addiction: The treatment effects of an REBT intervention for females. *Psychology of Sport and Exercise*, 64, 102298. 10.1016/j.psychsport.2022.102298 https://doi.org/10.1016/j.psychsport.2022.102298 37665799

[bib229] Kotbagi, G., Kern, L., Romo, L., & Pathare, R. (2015). The hierarchical model of exercise dependence: The development of the problematic practice of physical exercise scale. *Journal of Individual Differences*, 36(4), 247–257. 10.1027/1614-0001/a000172 https://doi.org/10.1027/1614-0001/a000172

[bib230] Kovacsik, R., Griffiths, M. D., Pontes, H. M., Soós, I., de la Vega, R., Ruíz-Barquín, R., Demetrovics, Z., & Szabo, A. (2018). The role of passion in exercise addiction, exercise volume, and exercise intensity in long-term exercisers. *International Journal of Mental Health and Addiction*, 17(6), 1389–1400. 10.1007/s11469-018-9880-1 https://doi.org/10.1007/s11469-018-9880-1

[B117] Krishnan, A. (2024). Integrative treatment for substance use disorders: Improving outcomes through evidence-based practice of yoga-derived breathwork and meditation. *Journal of Addiction Medicine*, 18(2), 103–109. 10.1097/ADM.0000000000001263 https://doi.org/10.1097/ADM.0000000000001263 38258889

[B118] Kun, B., Urbán, R., Szabo, A., Magi, A., Eisinger, A., & Demetrovics, Z. (2022). Emotion dysregulation mediates the relationship between psychological distress, symptoms of exercise addiction and eating disorders: A largescale survey among fitness center users. *Sport, Exercise, and Performance Psychology*, 11(2), 198–213. 10.1037/spy0000274 https://doi.org/10.1037/spy0000274

[B119] Landolfi, E. (2013). Exercise addiction. *Sports Medicine*, 43, 111–119. 10.1007/s40279-012-0013-x https://doi.org/10.1007/s40279-012-0013-x 23329605

[B120] Lassner, A., Papazova, I., Pross, B., Scherr, J., Schoenfeld, J., Halle, M., … Roeh, A. (2022). Exercise addiction measured at a naturalistic marathon-event–associations of the EAI with the general level of functioning, affect and performance parameters. *International Journal of Sport and Exercise Psychology*, 21(6), 1041–1053. 10.1080/1612197X.2022.2098357 https://doi.org/10.1080/1612197X.2022.2098357

[B121] Lichtenstein, M. B., Griffiths, M. D., Hemmingsen, S. D., & Støving, R. K. (2018). Exercise addiction in adolescents and emerging adults – Validation of a youth version of the Exercise Addiction Inventory. *Journal of Behavioral Addictions*, 7(1), 117–125. 10.1556/2006.7.2018.01 https://doi.org/10.1556/2006.7.2018.01 29409340 PMC6035018

[B123] Lichtenstein, M. B., Jensen, E. S., Larsen, P. V., Omdahl, M. K., & Szabo, A. (2020). Passion for exercise has three dimensions: Psychometric evaluation of the Passion Scale in a Danish fitness sample. *Translational Sports Medicine*, 3(6), 638–648. 10.1002/tsm2.173 https://doi.org/10.1002/tsm2.173

[B124] Lichtenstein, M. B., Melin, A. K., Szabo, A., & Holm, L. (2021). The prevalence of exercise addiction symptoms in a sample of national level elite athletes. *Frontiers in Sports and Active Living*, 3, 635418. 10.3389/fspor.2021.635418 https://doi.org/10.3389/fspor.2021.635418 34179773 PMC8222598

[B125] Lim, R. G., Voon, S. P., Yahya, F., Mohamad, F. S., & Ahmi, A. (2024). Global and LMIC insights into acceptance and commitment therapy (ACT): A bibliometric study from 1998 to 2023. *Journal of Contextual Behavioral Science*, 33, 100796. 10.1016/j.jcbs.2024.100796 https://doi.org/10.1016/j.jcbs.2024.100796

[B126] Linden, D. J. (n.d.). The truth behind ‘Runner’s High' and other mental benefits of running. *Johns Hopkins Medicine*, Retrieved March 25, 2025, from https://www.hopkinsmedicine.org/health/wellness-and-prevention/the-truth-behind-runners-high-and-other-mental-benefits-of-runni*ng.

[bib219] Loumidis, K. S., & Wells, A. (1998). Assessment of beliefs in exercise dependence: The development and preliminary validation of the Exercise Beliefs Questionnaire. *Personality and Individual Differences*, 25(3), 553–567. 10.1016/S0191-8869(98)00103-2 https://doi.org/10.1016/S0191-8869(98)00103-2

[B127] Maraz, A., Urbán, R., Griffiths, M. D., & Demetrovics, Z. (2015). An empirical investigation of dance addiction. *Plos One*, 10(5), e0125988. 10.1371/journal.pone.0125988 https://doi.org/10.1371/journal.pone.0125988 25951077 PMC4423970

[B128] Marazziti, D., Presta, S., Baroni, S., Silvestri, S., & Dell’Osso, L. (2014). Behavioral addictions: A novel challenge for psychopharmacology. *CNS Spectrums*, 19(5), 486–495. 10.1017/S1092852913000957 https://doi.org/10.1017/S1092852913000957 24589040

[B129] Marshall, L., & Goda, N. (2024). *CUriosity: What causes the runner’s high? CU Boulder today*. October 23. Retrieved December 10, 2024, from https://www.colorado.edu/today/2024/10/23/curiosity-what-causes-runners-high.

[B130] Mayolas-Pi, C., Sitko, S., Pano-Rodriguez, A., Lopez-Laval, I., Reverter-Masia, J., & Legaz-Arrese, A. (2025). Exercise addiction and psychosocial health risks among adolescent athletes: Focus on sport type and performance level. *Journal of Behavioral Addictions*, 14(2), 1095–1106. 10.1556/2006.2025.00024 https://doi.org/10.1556/2006.2025.00024 40168079 PMC12231450

[B131] McCabe, M. P., & Vincent, M. A. (2002). Development of body modification and excessive exercise scales for adolescents. *Assessment*, 9(2), 131–141. 10.1177/10791102009002003 https://doi.org/10.1177/10791102009002003 12066827

[B132] McElroy, S. L., Satlin, A., Pope, H. G., Keck, P. E., & Hudson, J. (1991). Treatment of compulsive shopping with antidepressants: A report of three cases. *Annals of Clinical Psychiatry*, 3(3), 199–204. 10.3109/10401239109148218 https://doi.org/10.3109/10401239109148218

[B133] McGlynn, F*. D. (2010). Systematic desensitization. In I. B. Weiner, & W. E. Craighead (Eds.), *Corsini encyclopedia of psychology* (pp. 755–764). John Wiley & Sons.

[B134] McNamara, J., & McCabe, M. P. (2012). Striving for success or addiction? Exercise dependence among elite Australian athletes. *Journal of Sports Sciences*, 30(8), 755–766. 10.1080/02640414.2012.667879 https://doi.org/10.1080/02640414.2012.667879 22420455

[B135] McNamara, J., & McCabe, M. P. (2013). Development and validation of the Exercise Dependence and Elite Athletes Scale. *Performance Enhancement & Health*, 2(1), 30–36. 10.1016/j.peh.2012.11.001 https://doi.org/10.1016/j.peh.2012.11.001

[B136] Melero Ventola, A. R., Yela, J. R., Crego, A., & Ortés-Rodríguez, M. (2020). Effectiveness of a mindfulness-based cognitive therapy group intervention in reducing gambling-related craving. *Journal of Evidence-Based Psychotherapies*, 20(1), 107–134. 10.24193/jebp.2020.1.7 https://doi.org/10.24193/jebp.2020.1.7

[B137] Meyer, M., Sattler, I., Schilling, H., Lang, U. E., Schmidt, A., Colledge, F., & Walter, M. (2021). Mental disorders in individuals with exercise addiction – A cross-sectional study. *Frontiers in Psychiatry*, 12, 751550. 10.3389/fpsyt.2021.751550 https://doi.org/10.3389/fpsyt.2021.751550 34955915 PMC8695763

[B138] Meyer, M., Wagner, A., Schmidt, A., Schaub, A. C., Lang, U. E., Walter, M., & Colledge, F. (2025). Stability of exercise addiction symptoms and co-occurring mental disorders–a follow-up study. *Frontiers in Psychiatry*, 16, 1494309. 10.3389/fpsyt.2025.1494309 https://doi.org/10.3389/fpsyt.2025.1494309 40212836 PMC11983396

[B139] Minutillo, A., Di Trana, A., Aquilina, V., Ciancio, G. M., Berretta, P., & La Maida, N. (2024). Recent insights in the correlation between social media use, personality traits and exercise addiction: A literature review. *Frontiers in Psychiatry*, 15, 1392317. 10.3389/fpsyt.2024.1392317 https://doi.org/10.3389/fpsyt.2024.1392317 38800058 PMC11116774

[bib227] Modolo, V. B., Antunes, H. K. M., de Gimenez, Santiago, M. L. D. M., Tufik, S., & de Mello, M. T. (2011). Negative addiction to exercise: Are there differences between genders? *Clinics*, 66(2), 255–260. 10.1590/S1807-59322011000200013 https://doi.org/10.1590/S1807-59322011000200013 21484043 PMC3059877

[B140] Mohanty, S., & Mitra, A. (2024). Yoga-meditation practice: A bridge to students’ good mental health. In D. Mishra, & J. D. Long (Eds.), *A pragmatic approach to religion and sustainability* (pp. 237–248). Nature Switzerland: Springer. 10.1007/978-3-031-67360-3_21 https://doi.org/10.1007/978-3-031-67360-3_21

[B141] Mónok, K., Berczik, K., Urbán, R., Szabo, A., Griffiths, M. D., Farkas, J., … Demetrovics, Z. (2012). Psychometric properties and concurrent validity of two exercise addiction measures: A population wide study. *Psychology of Sport and Exercise*, 13(6), 739–746. 10.1016/j.psychsport.2012.05.003 https://doi.org/10.1016/j.psychsport.2012.05.003

[B142] Monteiro, B., Galhardo, A., Senra, H., Pinto‐Gouveia, J., & Cunha, M. (2024). Beyond fight or flight: The protective role of pre‐pandemic meditation practice against anxiety and perceived stress. *Stress and Health*, 40(5), e3440. 10.1002/smi.3440 https://doi.org/10.1002/smi.3440 38953863

[B143] Morgan, W. P. (1979). Negative addiction in runners. *The Physician and Sports Medicine*, 7, 57–70. 10.1080/00913847.1979.11948436 https://doi.org/10.1080/00913847.1979.11948436 29256731

[bib222] Morgan, W. P., Costill, D. L., Flynn, M. G., Raglin, J. S., & O’connor, P. J. (1988). Mood disturbance following increased training in swimmers. *Medicine & Science in Sports & Exercise*, 20(4), 408–414. 10.1249/00005768-198808000-00014 https://doi.org/10.1249/00005768-198808000-00014 3173050

[B144] Nogueira, A., Molinero, O., Salguero, A., & Márquez, S. (2018). Exercise addiction in practitioners of endurance sports: A literature review. *Frontiers in Psychology*, 9, 1484. 10.3389/fpsyg.2018.01484 https://doi.org/10.3389/fpsyg.2018.01484 30174636 PMC6107830

[bib220] Ogden, J., Veale, D., & Summers, Z. (1997). The development and validation of the Exercise Dependence Questionnaire. *Addiction Research*, 5(4), 343–356. 10.3109/16066359709004348 https://doi.org/10.3109/16066359709004348

[B145] Olave, L., Iruarrizaga, I., Macía, P., Momeñe, J., Estévez, A., Muñiz, J. A., & Peñacoba, C. (2025). Exploring exercise addiction, self-esteem, and early maladaptive schemas: A cross-sectional study among female university students. *Healthcare*, 13(4), 422. 10.3390/healthcare13040422 https://doi.org/10.3390/healthcare13040422 39997297 PMC11854997

[B146] Paliliunas, D., Belisle, J., & Dixon, M. R. (2018). A randomized control trial to evaluate the use of acceptance and commitment therapy (ACT) to increase academic performance and psychological flexibility in graduate students. *Behavior Analysis in Practice*, 11(3), 241–253. 10.1007/s40617-018-0252-x https://doi.org/10.1007/s40617-018-0252-x 30363765 PMC6182845

[B147] Pallanti, S., Quercioli, L., Sood, E., & Hollander, E. (2002). Lithium and valproate treatment of pathological gambling: A randomized single-blind study. *Journal of Clinical Psychiatry*, 63(6), 559–566. 10.4088/JCP.v63n0617 https://doi.org/10.4088/JCP.v63n0617 12143910

[B209] Pasman, L. N., & Thompson, J. K. (1988). Body image and eating disturbance in obligatory runners, obligatory weightlifters, and sedentary individuals. *International Journal of Eating Disorders*, 7(6), 759–769.

[B148] Pittenger, C. (2015). Glutamate modulators in the treatment of obsessive-compulsive disorder. *Psychiatric Annals*, 45(6), 308–315. 10.3928/00485713-20150604-04 https://doi.org/10.3928/00485713-20150604-04 26236057 PMC4517847

[bib210] Plateau, C. R., Shanmugam, V., Duckham, R. L., Goodwin, H., Jowett, S., Brooke-Wavell, K. S., Laybourne, A., Arcelus, J., & Meyer, C. (2014). Use of the compulsive exercise test with athletes: Norms and links with eating psychopathology. *Journal of Applied Sport Psychology*, 26(3), 287–301. 10.1080/10413200.2013.867911 https://doi.org/10.1080/10413200.2013.867911

[B149] Popat, P., Dinu, L. M., Runswick, O., Findon, J. L., & Dommett, E. J. (2021). Investigating the relationship between attention-deficit hyperactivity disorder, obligatory exercise and exercise addiction. *International Journal of Mental Health and Addiction*, 21, 1365–1377. 10.1007/s11469-021-00662-4 https://doi.org/10.1007/s11469-021-00662-4

[B150] Potenza, M. N. (2017). Clinical neuropsychiatric considerations regarding nonsubstance or behavioral addictions. *Dialogues in Clinical Neuroscience*, 19(3), 281–291. 10.31887/DCNS.2017.19.3/mpotenza https://doi.org/10.31887/DCNS.2017.19.3/mpotenza 29302225 PMC5741111

[B151] Quesnel, D. A., Cooper, M., Cook, B., & Calogero, R. M. (2025). Evaluating the impact of a safe exercise training on clinician knowledge and self‐efficacy in managing dysfunctional exercise in an eating disorder treatment setting. *Journal of Evaluation in Clinical Practice*, 31(1), e14319. 10.1111/jep.14319 https://doi.org/10.1111/jep.14319 39868638

[B152] Ray, S., Bhanji, J., Kennelly, N., Fox, H. C., Budsock, P. D., Delgado, M., … Garland, E. L. (2024). Mindfulness-oriented recovery enhancement in opioid use disorder: Extended emotional regulation and neural effects and immediate effects of guided meditation in a pilot sample. *Explore*, 20(3), 434–438. 10.1016/j.explore.2023.11.001 https://doi.org/10.1016/j.explore.2023.11.001 37949774

[bib225] Rudy, E. B., & Estok, P. J. (1989). Measurement and significance of negative addiction in runners. *Western Journal of Nursing Research*, 11(5), 548–558. 10.1177/019394598901100504 https://doi.org/10.1177/019394598901100504 2815723

[B153] Ruegsegger, G. N., & Booth, F. W. (2018). Health benefits of exercise. *Cold Spring Harbor perspectives in medicine*, 8(7), a029694. 10.1101/cshperspect.a029694 https://doi.org/10.1101/cshperspect.a029694 28507196 PMC6027933

[B154] Sachs, M. L., & Pargman, D. (1979). Running addictions: A depth interview approach. *Journal of Sport Behavior*, 2, 143–155.

[B210] Samanta, P., Mohapatra, I., Mitra, R., Mishra, J., Mahapatra, P., Mohakud, N. K., Pattnaik, J. I., Behera, M. R., & Nanda, P. (2024). Mindfulness as a path to freedom from internet addiction in adolescents: A narrative review. *Cureus*, 16(10), e72544. 10.7759/cureus.72544 https://doi.org/10.7759/cureus.72544 39606537 PMC11600986

[B155] Sancho, M., De Gracia, M., Rodriguez, R. C., Mallorquí-Bagué, N., Sánchez-González, J., Trujols, J., … Menchón, J. M. (2018). Mindfulness-based interventions for the treatment of substance and behavioral addictions: A systematic review. *Frontiers in Psychiatry*, 9, 95. 10.3389/fpsyt.2018.00095 https://doi.org/10.3389/fpsyt.2018.00095 29651257 PMC5884944

[B156] Schaub, A., Meyer, M., Tschopp, A., Wagner, A., Lang, U. E., Walter, M., … Schmidt, A. (2024). Brain alterations in individuals with exercise dependence: A multimodal neuroimaging investigation. *Journal of Behavioral Addictions*, 13(2), 565–575. 10.1556/2006.2024.00028 https://doi.org/10.1556/2006.2024.00028 38842943 PMC11220813

[B157] Schaumberg, K., Bulik, C. M., & Micali, N. (2023). Patterns of maladaptive exercise behavior from ages 14–24 in a longitudinal cohort. *Journal of Child Psychology and Psychiatry*, 64(11), 1555–1568. 10.1111/jcpp.13844 https://doi.org/10.1111/jcpp.13844 37258173 PMC10592554

[B158] Schaumberg, K., Pictor, L., & Frank, M. (2024). Adaptive and maladaptive exercise in eating disorders. In J. M. Cisler, K. M. Crombie, & T. G. Adams (Eds.), *Exercise and mental health* (pp. 1–18). Springer. 10.1007/7854_2024_499 https://doi.org/10.1007/7854_2024_499 39042250

[B159] Shirk, S. D., Muquit, L. S., Deckro, J., Sweeney, P. J., & Kraus, S. W. (2022). Mindfulness-based relapse prevention for the treatment of gambling disorder among US military veterans: Case series and feasibility. *Clinical Case Studies*, 21(1), 3–17. 10.1177/15346501211020122 https://doi.org/10.1177/15346501211020122

[B160] Sicilia, A., Alcaraz-Ibáñez, M., Dumitru, D. C., Paterna, A., & Griffiths, M. D. (2020). Fitness- related self-conscious emotions and risk for exercise addiction: Examining the mediating role of passion. *Journal of Sport & Exercise Psychology*, 42(3), 240–248. 10.1123/jsep.2019-0260 https://doi.org/10.1123/jsep.2019-0260 32473581

[B161] Sicilia, Á., Alcaraz-Ibáñez, M., Paterna, A., & Griffiths, M. D. (2022). A review of the components of problematic exercise in psychometric assessment instruments. *Frontiers in Public Health*, 10, 839902. 10.3389/fpubh.2022.839902 https://doi.org/10.3389/fpubh.2022.839902 35433585 PMC9008204

[B162] Sicilia, A., Alcaraz-Ibáñez, M., Paterna, A., & Griffiths, M. D. (2023). Exercise addiction: Evolution and challenges for its recognition as a clinical disorder. In O. Corazza, & A. , R. Dores (Eds.), *The body in the mind: Exercise addiction, body image and the use of enhancement drugs* (pp. 1–24). Cambridge University Press. 10.1017/9781911623731.002 https://doi.org/10.1017/9781911623731.002

[B163] Sicilia, A., Paterna, A., Alcaraz-Ibanez, M., & Griffiths, M. D. (2021). Theoretical conceptualisations of problematic exercise in psychometric assessment instruments: A systematic review. *Journal of Behavioral Addictions*, 10(1), 4–20. 10.1556/2006.2021.00019 https://doi.org/10.1556/2006.2021.00019 33822749 PMC8969858

[bib218] Smith, D. K., Hale, B. D., & Collins, D. (1998). Measurement of exercise dependence in bodybuilders. *The Journal of Sports Medicine and Physical Fitness*, 38(1), 66–74. 9638035

[B164] Solmi, M., Basadonne, I., Bodini, L., Rosenbaum, S., Schuch, F. B., Smith, L., … Fusar-Poli, P. (2025). Exercise as a transdiagnostic intervention for improving mental health: An umbrella review. *Journal of Psychiatric Research*, 184, 91–101. 10.1016/j.jpsychires.2025.02.024 https://doi.org/10.1016/j.jpsychires.2025.02.024 40043589

[B165] Steffen, J. J., & Brehm, B. J. (1999). The dimensions of obligatory exercise. *Eating Disorders*, 7(3), 219–226. 10.1080/10640269908249287 https://doi.org/10.1080/10640269908249287

[B166] Stein, D. J., Hollander, E., Anthony, D. T., Schneier, F. R., Fallon, B. A., Liebowitz, M. R., & Klein, D. F. (1992). Serotonergic medications for sexual obsessions, sexual addiction, and paraphilias. *Journal of Clinical Psychiatry*, 53(7), 267–271. 1386848

[B167] Stöver, H. (2011). Barriers to opioid substitution treatment access, entry and retention: A survey of opioid users, patients in treatment, and treating and non-treating physicians. *European Addiction Research*, 17(1), 44–54. 10.1159/000320576 https://doi.org/10.1159/000320576 20975276

[B168] Strahler, J., Wachten, H., Stark, R., & Walter, B. (2021). Alike and different: Associations between orthorexic eating behaviors and exercise addiction. *International Journal of Eating Disorders*, 54(8), 1415–1425. 10.1002/eat.23525 https://doi.org/10.1002/eat.23525 33955559

[B169] Sukhera, J. (2022). Narrative reviews: Flexible, rigorous, and practical. *Journal of Graduate Medical Education*, 14(4), 414–417. 10.4300/JGME-D-22-00480.1 https://doi.org/10.4300/JGME-D-22-00480.1 35991099 PMC9380636

[B170] Sussman, S., Leventhal, A., Bluthenthal, R. N., Freimuth, M., Forster, M., & Ames, S. L. (2011). A framework for the specificity of addictions. *International Journal of Environmental Research and Public Health*, 8(8), 3399–3415. 10.3390/ijerph8083399 https://doi.org/10.3390/ijerph8083399 21909314 PMC3166750

[bib209] Swenne, I. (2016). Evaluation of the compulsive exercise test (CET) in adolescents with eating disorders: Factor structure and relation to eating disordered psychopathology. *European Eating Disorders Review*, 24(4), 334–340. 10.1002/erv.2439 https://doi.org/10.1002/erv.2439 26892035

[B171] Szabo, A. (1995). The impact of exercise deprivation on well-being of habitual exercisers. *The Australian Journal of Science and Medicine in Sport*, 27, 68–75. 8599747

[B172] Szabo, A. (2001, May). The dark side of sports and exercise: Research dilemmas. In *Paper presented at the 10th world congress of sport psychology*. Skiathos, Greece.

[B173] Szabo, A. (2010). *Addiction to exercise: A symptom or a disorder?* Nova Science Publishers.

[B174] Szabo, A. (2018). Addiction, passion, or confusion? New theoretical insights on exercise addiction research from the case study of a female body builder. *Europe's Journal of Psychology*, 14(2), 296. 10.5964/ejop.v14i2.1545 https://doi.org/10.5964/ejop.v14i2.1545 PMC6016027 30008948

[B175] Szabo, A. (2024). Chasing a phantom dysfunction: A position paper on current methods in exercise addiction research. *International Journal of Mental Health and Addiction**,* 23, 4600–4611. 10.1007/s11469-024-01372-3 https://doi.org/10.1007/s11469-024-01372-3

[B176] Szabo, A., de la Vega, R., Kovácsik, R., Jiménez Almendros, L., Ruíz-Barquín, R., Demetrovics, Z., … Köteles, F. (2022). Dimensions of passion and their relationship to the risk of exercise addiction: Cultural and gender differences. *Addictive Behaviors Reports*, 16, 100451. 10.1016/j.abrep.2022.100451 https://doi.org/10.1016/j.abrep.2022.100451 36092546 PMC9450070

[B177] Szabo, A., & Demetrovics, Z. (2022). *Passion and addiction in sports and exercise*. Routledge. 10.4324/9781003173595 https://doi.org/10.4324/9781003173595

[B178] Szabo, A., Griffiths, M. D., de La Vega Marcos, R., Mervo, B., & Demetrovics, Z. (2015). Methodological and conceptual limitations in exercise addiction research. *Yale Journal of Biology and Medicine*, 3(88), 303–308. 10.1249/00005768-198704000-00005 https://doi.org/10.1249/00005768-198704000-00005 PMC4553651 26339214

[B179] Szabo, A., Griffiths, M. D., & Demetrovics, Z. (2019). Psychology and exercise. In D. Bagchi, S. Nair, & C. K. Sen (Eds.), *Nutrition and enhanced sports performance* (2nd ed., pp. 63–72). Academic Press. 10.1016/B978-0-12-813922-6.00005-9 https://doi.org/10.1016/B978-0-12-813922-6.00005-9

[B180] Szabo, A., & Kovacsik, R. (2019). When passion appears, exercise addiction disappears. *Swiss Journal of Psychology*, 78(3–4), 137–142. 10.1024/1421-0185/a000228 https://doi.org/10.1024/1421-0185/a000228

[B181] Szabo, A., Pinto, A., Griffiths, M. D., Kovacsik, R., & Demetrovics, Z. (2019). The psychometric evaluation of the Revised Exercise Addiction Inventory: Improved psychometric properties by changing item response rating. *Journal of Behavioral Addictions*, 8(1), 157–161. 10.1556/2006.8.2019.06 https://doi.org/10.1556/2006.8.2019.06 30920295 PMC7044604

[B182] Szabo, A., Tóth, E., Kósa, L., Laki, Á., & Ihász, F. (2021). Increased exercise effort after artificially-induced stress: Laboratory-based evidence for the catharsis theory of stress. *Baltic Journal of Sport and Health Sciences*, 4(119), 24–30. 10.33607/bjshs.v4i119.1016 https://doi.org/10.33607/bjshs.v4i119.1016

[bib213] Taranis, L., Touyz, S., & Meyer, C. (2011). Disordered eating and exercise: Development and preliminary validation of the Compulsive Exercise Test (CET). *European Eating Disorders Review*, 19(3), 256–268. 10.1002/erv.1108 https://doi.org/10.1002/erv.1108 21584918

[B183] Terry, A., Szabo, A., & Griffiths, M. D. (2004). The Exercise Addiction Inventory: A new brief screening tool. *Addiction Research and Theory*, 12(5), 489–499. 10.1080/16066350310001637363 https://doi.org/10.1080/16066350310001637363

[B184] Tharumiya, A. K., Riniprabha, P., Sakthivel, K., Janani, K., & Manicka, M. M. (2024). Influence of mindfulness on game addiction - Mediating role of emotional control. *Psychological Reports*, 129(1), 63–74. 10.1177/00332941241232940 https://doi.org/10.1177/00332941241232940 38340087

[B185] Thompson, J. K., & Blanton, P. (1987). Energy conservation and exercise dependence: A sympathetic arousal hypothesis. *Medicine and Science in Sports and Exercise*, 19, 91–97. 10.1249/00005768-198704000-00005 https://doi.org/10.1249/00005768-198704000-00005 3574055

[B186] Tran, M. A. Q., Vo-Thanh, T., Soliman, M., Ha, A. T., & Van Pham, M. (2024). Could mindfulness diminish mental health disorders? The serial mediating role of self-compassion and psychological well-being. *Current Psychology*, 43(15), 13909–13922. 10.1007/s12144-022-03421-3 https://doi.org/10.1007/s12144-022-03421-3 PMC9362435 35967505

[B187] Trott, M., Jackson, S. E., Firth, J., Jacob, L., Grabovac, I., Mistry, A., … Smith, L. (2021). A comparative meta-analysis of the prevalence of exercise addiction in adults with and without indicated eating disorders. *Eating and Weight Disorders-Studies on Anorexia, Bulimia and Obesity*, 26, 37–46. 10.1007/s40519-019-00842-1 https://doi.org/10.1007/s40519-019-00842-1 31894540

[B188] Trott, M., Johnstone, J., McDermott, D. T., Mistry, A., & Smith, L. (2022). The development and validation of the Secondary Exercise Addiction Scale. *Eating and Weight Disorders-Studies on Anorexia, Bulimia and Obesity*, 27(4), 1427–1436. 10.1007/s40519-021-01284-4 https://doi.org/10.1007/s40519-021-01284-4 34370271

[B189] Tuttle, L. W. (1992). *Compulsive exercise: The symptomatology, associated psychopathology, and diagnostic validity of a compulsive exercise syndrome*. PhD Dissertation. New School for Social Research, New York, United States.

[B190] van der Tempel, J., McDermott, K., Niepage, M., Afifi, T. O., McMain, S., Jindani, F., … Zack, M. (2020). Examining the effects of mindfulness practice and trait mindfulness on gambling symptoms in women with gambling disorder: A feasibility study. *International Gambling Studies*, 20(1), 114–134. 10.1080/14459795.2019.1686766 https://doi.org/10.1080/14459795.2019.1686766

[bib231] Veale, D. M. W. (1987). Exercise dependence. *British Journal of Addiction*, 82(7), 735–740. 10.1111/j.1360-0443.1987.tb01539.x https://doi.org/10.1111/j.1360-0443.1987.tb01539.x 3311101

[B191] Verhaeghen, P. (2021). Mindfulness as attention training: Meta-analyses on the links between attention performance and mindfulness interventions, long-term meditation practice, and trait mindfulness. *Mindfulness*, 12(3), 564–581. 10.1007/s12671-020-01532-1 https://doi.org/10.1007/s12671-020-01532-1

[B192] Vítková, T., Rusnáková, K., & Mudrák, J. (2025). Personality predictors of exercise addiction in competitive sport. *Journal of Sports Sciences*, 43(9), 865–874. 10.1080/02640414.2025.2477922 https://doi.org/10.1080/02640414.2025.2477922 40091662

[B193] Wang, Y., Hua, G., Liu, W., Wan, C., Hao, M., & Zhang, M. (2025). Exercise addiction in college students: The impact of body dissatisfaction, stress, physical activity and gender. *Frontiers in Psychiatry*, 16, 1546192. 10.3389/fpsyt.2025.1546192 https://doi.org/10.3389/fpsyt.2025.1546192 40071282 PMC11893607

[B194] Wang, Y., Shi, H., Liu, S., Wang, K., Griffiths, M. D., & Szabo, A. (2024). Psychometric evaluation of the Revised Exercise Addiction Inventory (EAI-R) among Chinese college students. *International Journal of Mental Health and Addiction*, 22(4), 1743–1760. 10.1007/s11469-022-00955-2 https://doi.org/10.1007/s11469-022-00955-2

[B195] Warburton, D. E., & Bredin, S. S. (2019). Health benefits of physical activity: A strengths-based approach. *Journal of Clinical Medicine*, 8(12), 2044. 10.3390/jcm8122044 https://doi.org/10.3390/jcm8122044 31766502 PMC6947527

[B196] Weiermair, T., Svehlikova, E., Boulgaropoulos, B., Magnes, C., & Eberl, A. (2024). Investigating runner’s high: Changes in mood and endocannabinoid concentrations after a 60 min outdoor run considering sex, running frequency, and age. *Sports*, 12(9), 232. 10.3390/sports12090232 https://doi.org/10.3390/sports12090232 39330709 PMC11435531

[B197] Weinstein, A., & Szabo, A. (2023). Exercise addiction: A narrative overview of research issues. *Dialogues in Clinical Neuroscience*, 25(1), 1–13. 10.1080/19585969.2023.2164841 https://doi.org/10.1080/19585969.2023.2164841 36698618 PMC9869993

[B198] Weinstein, A., & Weinstein, Y. (2014). Exercise addiction-diagnosis, bio-psychological mechanisms and treatment issues. *Current Pharmaceutical Design*, 20(25), 4062–4069. 10.2174/13816128113199990614 https://doi.org/10.2174/13816128113199990614 24001300

[B199] Winiarz, E. (2019). Endorphins, endocannabinoids and runners’ high. *The Science Journal of the Lander College of Arts and Sciences*, 13(1), 24–33.

[B200] World Health Organization. (2019). *International statistical classification of diseases and related health problems*. Retrieved March 25, 2025, from https://www.who.int/standards/classifications/classification-of-diseases.

[B201] World Health Organization. (2023). *Global recommendations on physical activity for health*. Retrieved March 25, 2025, from https://www.who.int/publications/i/item/9789241599979. 26180873

[B202] World Health Organization. (2024). *Physical activity*. Retrieved March 25, 2025, from https://www.who.int/news-room/fact-sheets/detail/physical-activity.

[B203] Wu, R., Jing, L., Liu, Y., Wang, H., Xie, L., & Deng, W. (2023). Effects of mindfulness on obligatory exercise during the return of injured athletes to sports: The mediating roles of self-criticism and competitive state anxiety. *Psychology Research and Behavior Management*, 16, 2157–2171. 10.2147/PRBM.S414709 https://doi.org/10.2147/PRBM.S414709 37334404 PMC10274843

[B204] Xie, H., Zhang, F., Gan, S., Wu, J., Wu, B., Qin, K., … Jia, Z. (2024). Body satisfaction, exercise dependence, and white matter microstructure in young adults. *Journal of Magnetic Resonance Imaging*, 61(2), 749–755. 10.1002/jmri.29485 https://doi.org/10.1002/jmri.29485 38874990

[bib223] Yates, A., Leehey, K., & Shisslak, C. M. (1983). Running—An analogue of anorexia? *New England Journal of Medicine*, 308(5), 251–255. 6848935 10.1056/NEJM198302033080504

[B206] Zimmerman, M., Breen, R. B., & Posternak, M. A. (2002). An open-label study of citalopram in the treatment of pathological gambling. *Journal of Clinical Psychiatry*, 63(1), 44–48. 10.4088/JCP.v63n0111 https://doi.org/10.4088/JCP.v63n0111 11838625

[B207] Zinn, J. (2003). Mindfulness-based interventions in context: Past, present, and future. *Clinical Psychology: Science and Practice*, 10(2), 144–156. 10.1093/clipsy.bpg016 https://doi.org/10.1093/clipsy.bpg016

